# Affective Robotics: Modelling and Testing Cultural Prototypes

**DOI:** 10.1007/s12559-014-9299-3

**Published:** 2014-08-14

**Authors:** Paul A. Wilson, Barbara Lewandowska-Tomaszczyk

**Affiliations:** University of Lodz, Lodz, Poland

**Keywords:** Affective robotics, Body features of emotion, Corpus materials, Culture prototypes, Emotion event scenario, GRID

## Abstract

If robots are to successfully interact with humans, they need to measure, quantify and respond to the emotions we produce. Similar to humans, the perceptual cue inputs to any modelling that allows this will be based on behavioural expression and body activity features that are prototypical of each emotion. However, the likely employment of such robots in different cultures necessitates the tuning of the emotion feature recognition system to the specific feature profiles present in these cultures. The amount of tuning depends on the relative convergence of the cross-cultural mappings between the emotion feature profiles of the cultures where the robots will be used. The GRID instrument and the cognitive corpus linguistics methodology were used in a contrastive study analysing a selection of behavioural expression and body activity features to compare the feature profiles of joy, sadness, fear and anger within and between Polish and British English. The intra-linguistic differences that were found in the profile of emotion features suggest that weightings based on this profile can be used in robotic modelling to create emotion-sensitive socially interacting robots. Our cross-cultural results further indicate that this profile of features needs to be tuned in robots to make them emotionally competent in different cultures.

## Introduction

To accomplish successful social interaction with humans, robots need to identify and respond to emotions. In order to achieve human-like competence in decoding emotions, such robots would need to measure and quantify the same sensory cues that are processed by humans, namely linguistic, paralinguistic, facial, body movement and physiological features. One of the aims of the present study reported in this paper was to compare sadness, joy, fear and anger on such sensory cues within British English and within Polish. Generally recognised as basic emotions, joy, sadness, fear and anger were selected on the basis of being relatively common emotions that signify the occurrence of important events. It is such central emotions that emotion-sensitive socially interacting robots should be able to detect and respond to if they are to be emotionally competent socially interacting beings. Although there has been progress in research investigating robotic recognition and interpretation of sensory cues with regard to emotion identification, relatively little empirical attention has been focused on how socially interactive robots might overcome cross-cultural challenges in order to accurately decode emotions in different cultures. The second and main aim of our study was to investigate such challenges by assessing the differences between Polish and British English in the profiles of joy, sadness, fear and anger on linguistic, paralinguistic, facial, body movement and physiological features. Any socially interactive entity, be it a human or a robot, would need to distinguish between these differences to successfully decode emotions in the two cultures.

The wealth of recent studies showing cross-linguistic and cross-cultural differences in emotions highlights the challenge that emotion-sensitive interactive robots face if they are to interact socially in different languages and cultures. In our research employing the GRID (see below for more details) and corpus methodologies, we have observed, for example, that British English *fear* is more of an energising emotion than its Polish counterpart *strach*, suggesting that British English *fear* is more akin to a variant of fear in which an individual feels powerful and dominant and, if successful, is in control of fear [18]. We have also shown that Polish and English differ in their emotional profiles of happiness and contentment [37], and surprise [17]. Other studies have shown similar inequivalences in language terms across languages and cultures. For example, Alonso-Arbiol and van der Vijer [1] observed that whereas Spanish ‘*desesperaciόn*’ (despair) is a combination of sadness and the anger elements of frustration/exasperation, the English and Basque (‘*etsipena*’) equivalents are instances of a more general sadness category. Demonstrating cross-cultural emotion differences within one language, Mortillaro et al. [20] show that whereas Southern Italians conceptualise pride negatively, Northern Italians consider pride to be a comparably more positive emotion. The results of a study by Ishii [12] revealed that whereas happiness was associated more with pride in American English on bodily reaction features, it was more connected with love on these features in Japanese. The question that arises is whether such diverse cross-linguistic and cross-cultural differences have a theoretical explanation. Although some of the inequivalent cross-linguistic and cross-cultural emotion patterning is accounted for within the framework of Hofstede’s [11] collectivism versus individualism, other findings appear idiosyncratic to specific cultures and fall outside overarching theoretical explanation. The main aim of the present study was to assess the differences between Polish and British English in the expression feature profiles of joy, sadness, fear and anger that might be decoded by a socially competent robot, as it is these differences that any entity navigating within a socially interactive environment would need to identify in order to operate successfully at an emotional level. Therefore, the approach adopted in the present paper is pragmatic in the sense of identifying differences in emotion features that would allow robots to be linguistically and culturally competent in terms of emotion differences rather than identifying the underlying theoretical reasons for these differences.^1^

Our contribution to affective robotics in the present paper presupposes a theory of human emotions and includes their description and presentation. Social robotics considers emotions in their broad biological sense as *automated homoeostatic regulation* [5] at different levels of biological behaviour, including man–robot linguistic communication. Taken in this sense, robots, both expressing artificial emotions as well as perceiving and recognising them, can be modelled in terms of emotional-homoeostatic architecture. The research methodology applied can either be more directly applicable at the interface and robot design of emotion modelling or can provide some materials on which to further develop models of socially interactive robots to be used in different functions such as medical and therapeutic aids and educational and entertaining devices.

While preliminary emphasis in social robotics is conventionally put on dyadic query-response acts in man–machine communication, more sophisticated modelling will involve more varied signals (components) and patterns of linguistic structures and behaviour for emotion recognition and expression. A change of *events* in the surrounding is often signalled via (bodily and linguistic) emotional behaviour in human agents and is likely to be perceived as such by robots. In other words, human emotional behaviour, expressed by our bodies and language, signals changes in the contextual surrounding, and whereas some of these can be, for instance, threatening and induce fear, others will be entertaining and constitute antecedents of joy.

Our emotion research uses two approaches to emotion studies: the GRID and cognitive corpus linguistics methodologies. The GRID instrument [9, 26] employs a system of dimensions and components, which bring about insight into the nature of emotion prototypical structures. The GRID project is coordinated by the Swiss Center for Affective Sciences at the University of Geneva in collaboration with Ghent University and is a worldwide study of emotional patterning across 23 languages and 27 countries. The GRID instrument comprises a Web-based questionnaire in which 24 prototypical emotion terms are evaluated on 144 emotion features. These features represent activity in all six of the major components of emotion. Thirty-one features relate to *appraisals of events*, eighteen to *psychophysiological changes*, twenty-six to *facial, vocal or gestural* *expressions*, forty to *action tendencies*, twenty-two to *subjective experiences* and four to *emotion regulation*. An additional three features refer to other qualities, such as *frequency* and *social acceptability* of the emotion. Participants are asked to rate the likelihood of these features for the various emotions. This methodology is comprehensive in its scope as it allows the multicultural comparison of emotion conceptualisations on all six of the emotion categories recognised by emotion theorists [7, 21, 26].

The cognitive corpus linguistics approach provides information on the probabilities of the occurrence of some linguistic patterns of emotional language use based on their frequencies and distributional patterns. Both methods are employed in the context of basic emotion event scenarios (EES) (Lewandowska-Tomaszczyk and Wilson [19], which identify the effects of emotion stimuli on Experiencer, and his/her embodied (bodily and mental) and exbodied reactions, linguistically expressed via segmental and prosodic properties, which characterise some but not other emotional states. Our proposal of a Prototypical EES [36] covers the following constituents:

**Context** (Biological predispositions of Experiencer; Social and Cultural conditioning; On-line contextual properties of Event) [**Stimulus** **→** **Experiencer** {(internally and externally manifested) physiological and physical symptoms; affective state + (internally experienced) Emotion} **→** possible **external reaction(s)** of Experiencer (blending; **language**: metaphor; emotion and emotional talk; **non-verbal** reactions)]

Language corpora are large collections of language materials, both spoken and written, and are representative of different linguistic styles and genres. The corpus materials used in the analysis reported in the present paper are derived from the British National Corpus (BNC: http://www.natcorp.ox.ac.uk/ comprising 100 million words) and the National Corpus of Polish (nkjp.pl, comprising over 260 million words).

The internal structure of emotion concepts and the regularities in their co-occurrence with bodily and contextual emotion signals are also analysed, taking insight from cognitive linguistics [14], Langacker [15], particularly from the analysis of figurative (first of all, metaphoric) language. Metaphorisation is a mental process of perceiving one (usually more abstract) object or event in terms of another, which is typically a more concrete one.

Other relevant concepts in linguistics are collocations. A collocation [10] is a sequence of words or terms that co-occur more often than would be expected by chance. For example, the Polish phrase *wpaść w gniew* (lit. ‘fall in anger’) is a conventional (metaphoric) collocation in Polish while in English the expression? *fall in anger* would not be considered correct by native speakers of English; by contrast, the expression *fall in love* would be fully acceptable as an English collocation. The ‘*fall in*’ metaphors in these collocations highlight the fairly sudden, frequently unexpected and not fully controlled, nature of this manifestation of emotion. Another metaphoric collocation involving the descriptive element of *venting* (e.g. *he vented his embarrassment/anger/frustration by…*) underlies the negative character of these emotions and the process which leads to decreased power and weakening of the Experiencer, letting metaphorical air (anger) escape or release from confinement and, similarly to a tyre, making it flat (devoid of tension—power). Metaphors and the verbal material in collocations are retrievable from corpus materials. Research on corpus data uncovers elements of the EES and is likely to point to the consequences of relevant stimuli, and the properties of major significance in emotionally threatening contexts and their possible negative outcomes, which are crucial for social robotics.

The tools used to generate concordance and collocation sets for both languages are a set of HASK tools (Pelcra-Hask.pl), which display collocations in basic Part-Of-Speech patterns and their frequencies.^2^ Parts of speech are linguistic categories of words, which are characterised by similar (syntactic and morphological) properties and distribution in the sentence. The main parts of speech are Nouns (*boy, chair, water*, etc.), Adjectives (*tall, old, lovely*, etc.), Verbs (*dance, write, grow, be*, etc.) and Adverbs (*slowly, well*, *fast*, etc.).

Collocations with major parts of speech can be automatically generated from corpus data. Collocates of a word are generated in a window of 5 words before and after the investigated term, which is the standard practice in corpus linguistics, and the relevant *t* test scores are provided. Programs calculate the degree of association between terms based on measures of association, which, in our study, refers to the value of Mutual Information (MI). Our software also offers the possibility of filtering the collocations in terms of their grammatical class (Nouns, Verbs, Adjectives), which we find useful to identify in order to elaborate on the components of particular Emotion Scenarios (Experiencers, Stimuli/Sources, etc.). The top 15 collocations for each of the Polish and English emotions relevant to the present paper are presented in the tables in the appendices. Regular patterns of collocation structure, in which an emotion term is a headword that co-occurs with its closest collocates, uncover regularities in Polish and English emotion content and corresponding behavioural correlates.

Secondly, in addition to inspecting a list of lexical collocates of an emotion word (e.g. *fear*, *joy* or *sadness*), there is an added value in looking at the full verbal contexts in which the word is used because it can reveal additional characteristics of the emotion (for example, eliciting event sources and its expression). The analysis of the phrases and sentences in which a word appears can be done through Key Word in Context (KWIC) searches, where the analyst specifies the word to be looked up, and the program retrieves all instances of use of that word in the corpus and its immediate context. The lexical items immediately surrounding a headword are also referred to as the word’s *concordance.* In our methodology, additional properties of emotions generated from the corpora enrich and provide independent evidence for the characteristics obtained by means of the GRID questionnaire.

As observed in our previous studies [37], some emotions like *happiness* and *szczęście*, in some of their senses, display a more overlapping equivalent structure between English and Polish; however, some others (*fear—strach* and *sadness—smutek*) diverge to a larger extent. Additionally, there are emotions such as *anger* in English, which are conventionally correlated with two distinct counterparts in Polish, *gniew* and *złość* (see [33]; relevant lexicographic data is also available from bilingual dictionaries, e.g. *Great English*-*Polish Dictionary* 2002). In the present paper, we make an attempt to establish to what extent English *anger* is a blended combination of *gniew* and *złość*, and in which contexts its behaviour in language use can provide clues to identify its two senses: one more similar to *gniew*—in which ‘anger’ designates a more predictable and controllable emotion and the other resembling *złość*—an emotion more difficult to constrain.

Our system does not provide all the information to enable explicit, rich-context modelling of emotion production or perception, but provides sufficient data to model culture-bound human-like emotional behaviour by resorting to the clusters of preferential conditions present in the implementation of a particular EES (see above) in a given cultural and linguistic context. In this sense, our data should be viewed as complementing other studies focusing on the robotic encoding and decoding of emotions.

In this study, we focus on the expression characteristics of emotions. More specifically, we look at five types of perceptual features (or ‘sensory cues’) that signal the presence of an emotion: linguistic features (e.g. *produced a short utterance*), paralinguistic features (e.g. *had a trembling voice*), facial features (e.g. *frowned*), body movement features (e.g. *abrupt bodily movements*) and physiological features (e.g. *breathing getting faster*). Accurate decoding of such sensory cues is fundamental to the interactive success that social robots need to achieve when communicating with humans. The data from the five categories of sensory cues reported in the present study are relevant to the main fields of robotics emotion research. Wimmer et al. [38] report that, in a model with facial feature extraction comprising structural and facial features, robots are able to recognise 67 % of human facial expressions. Vogt et al. [30] outline solutions to the problems that prevent automatic emotion recognition systems being able to recognise human emotions from vocal cues in real time. Castellano et al. [4] demonstrate that ‘high’ and ‘low arousal’ emotions can be distinguished from ‘positive’ and ‘negative’ emotions from expressive motion cues that could be used by artificial, automatic systems to decode emotions from human movement. Barber et al. [2] assess the state of the art regarding the robotic remote encoding of human physiological measures in natural settings and the steps that are necessary for humans to use this information in decision making. The common fundamental element of these studies is the identification of the sensory cues that socially interactive robots need to accurately decode if they are to successfully distinguish between human emotions.

We aim to investigate sensory cue profiles in the present study to determine both intra-linguistic and inter-linguistic differences in emotion expression. Profiles of sensory cues have been employed effectively to determine conceptual differences between emotions within languages. For example, using the GRID methodology, Ogarkova et al. [22] analysed the means of sensory cue features (e.g. *showed tears*, *spoke slower*, *frowned*, *spoke faster* and *felt hot*) to show that Russian *toska* is conceptually closer to *sadness* than to *anxiety/fear*. Turning to a cross-linguistic perspective, Scherer and Walbott [27] employed a methodology in which participants from 37 countries were asked to provide physiological symptoms and expressive reactions, including bodily symptoms, non-verbal expressive reactions, and verbal reactions, to recalled, personal emotional situations. The relatively strong universal, emotion-specific effects showed differences in the profiles for joy, fear, anger, sadness, disgust, shame and guilt. It was observed, for example, that joy is characterised by very expressive non-verbal and verbal behaviour, a strong orientation towards other people and a feeling of warmth or heat. By contrast, sadness has a strong non-verbal expression but little vocal or verbal behaviour and is further characterised by an orientation away from other people and a feeling of being cold. Fear has a relatively lower outward expression, with high arousal and a feeling of coldness. Anger is characterised by high verbal and non-verbal expression, with high arousal and high felt temperature. These emotions were relatively consistent across the cultures examined, with only small to moderate interactions between country and emotion. However, it must be noted that the variables comprised the composite sum of sensory cue features. For example, the verbal behaviour variable was the sum of *silence*, *short utterance*, *one/two sentences* and *long utterance*. In comparison, more recent studies that have included a more fine-grained focus on specific sensory features in different perceptual modalities have shown more pronounced cross-cultural differences. Comparing Western Caucasian and East Asian observations of computer-modelled facial expressions of happiness, surprise, fear, disgust, anger and sadness, Jack et al. [13] concluded that “facial expressions of emotion are culture specific” (p. 7242). Laukka et al. [16] employed machine learning simulations to classify the vocal expression of emotions produced by professional actors in 5 English-speaking cultures. Although there was some cross-cultural consistency in the classifications of emotions, further results showed a within-culture recognition advantage of vocally expressed emotions in comparison with the cross-cultural condition. To conclude, the emotion-specific effects regarding the differences in sensory cues appear to be greater than the cross-cultural differences. However, there is evidence, especially in more recent studies, pointing to more pronounced differences in such cues across cultures.

## GRID Methodology

### Procedure

Participants completed the GRID instrument in a controlled Web study [25], in which each participant was presented with four emotion terms randomly chosen from the set of 24 and asked to rate each in terms of the 144 emotion features. They rated the likelihood that each of the 144 emotion features can be inferred when a person from their cultural group uses the emotion term to describe an emotional experience. A 9-point scale was employed that ranged from *extremely unlikely* (1) to *extremely likely* (9)—the numbers 2 to 8 were placed at equidistant intervals between the two ends of the scale, with 5 ‘neither unlikely, nor likely’ in the middle and participants typed their ratings on the keyboard. It was clearly stated that the participants needed to rate the likelihood of occurrence of each of the features when somebody who speaks their language describes an emotional experience with the emotion terms presented. Each of the 144 emotion features was presented separately, and participants rated all four emotion terms for that feature before proceeding to the next feature.

### Participants

The mean ages and gender ratios of the participants for each of the emotion terms were as follows: *joy* (35 British English-speaking participants; mean age 21.3 years, 21 females); *sadness* (33 British English-speaking participants; mean age 21.7 years, 19 females); *fear* (36 British English-speaking participants; mean age 21.5 years, 21 females); and *anger* (32 British English-speaking participants; mean age 20.8 years, 20 females). The mean ages and gender ratios of the participants for each of the emotion terms were as follows: *radość* (27 Polish-speaking participants; mean age 22.3 years, 16 females); *smutek* (22 Polish-speaking participants; mean age 23.6 years, 14 females); *strach* (32 Polish-speaking participants; mean age 23.4 years, 19 females); *złość* (25 Polish-speaking participants; mean age 22.5 years, 13 females); and *gniew* (31 Polish-speaking participants; mean age 27.2 years, 18 females).

### GRID Features and Emotions

The present study reported in this paper used thirty-three GRID features that were selected on the basis of behavioural expression or body activity and, apart from *sing and dance*, can be grouped into the following five categories: *physiological features*, *facial expression features*, *body movement features*, *paralinguistic features* and *linguistic features* (see Tables 1 and 2 for the selection of GRID features).

The emotions selected for the present study were British English *sadness*, *joy*, *fear* and *anger*, and their Polish counterparts *smutek*, *radość*, *strach*, *złość* and *gniew*, the latter two being common types of anger in Polish [33]. Anger was specifically chosen to highlight the complexity of cross-cultural differences that emotion-sensitive robots will need to address if they are to be used in different cultures.

Both *intra-*linguistic and English–Polish *inter-*linguistic differences are made more explicit in terms of the GRID components and in the corpus data as will be discussed in the sections to follow.

### GRID Dimensions

In an initial study of the dimensional structure of emotions using the GRID instrument, Fontaine et al. [8] derived a four-dimensional structure for English, French and Dutch that comprised *valence*, *power*, *arousal* and *novelty*. Analyses performed on the data from all of the languages represented in the GRID project have reproduced this dimensional structure [9]. To determine the dimensional structure of the Polish and British English data in the present study, principle components analysis (PCA) with varimax rotation was performed on the combined dataset of these two languages. There were 201 British English participants (124 females) with a men age of 21.5 years, and 124 Polish participants (95 females) with a mean age of 23.2 years. The four-dimensional solution that was selected comprised the same dimensions as Fontaine et al. [8] and Fontaine et al. [9] and accounted for 81.9 % of the total variance. The first dimension (*valence*) accounted for 52.9 % of the variance, the second dimension (*power*) for 15.5 %, the third dimension (*arousal*) for 8.3 % and the last dimension (*novelty*) for 5.1 %. A sensory cue GRID feature was included in a dimension if it achieved a 0.6 loading on this dimension (see "Appendix 2" for loadings of sensory cue GRID features on dimensions). The *valence* dimension is characterised by appraisals of intrinsic pleasure and goal conduciveness. Other features include action tendencies of approach versus avoidance, and pleasant emotions versus unpleasant emotions (*valence* sensory cue features in the present study: *felt cold*, *smiled*, *pressed lips together*, *frowned*, *moved towards people or things*, *withdrew from people or things*, *had a trembling voice*, *produced a short utterance*, *produced a long utterance*, *produced speech disturbances*, *wanted to sing or dance*). *Power* includes appraisals of control, leading to feelings of power and weakness. It is also characterised by appraisals of interpersonal dominance or submission (*power* sensory cue features in the present study: *closed his or her eyes*, *increased the volume of voice*, *decreased the volume of voice*, *had an assertive voice*, *fell silent*, *spoke faster*, *spoke slower*). The *arousal* dimension is mainly characterised by sympathetic arousal (*arousal* sensory cue features in the present study: *felt shivers*, *heartbeat slowing down*, *heartbeat getting faster*, *breathing slowing down*, *breathing getting faster*, *perspired/moist hands*, *sweat*, *felt hot*, *abrupt bodily movements*). The fourth dimension is represented by *novelty*. On this dimension, appraisals of novelty and unpredictability are compared with expectedness or familiarity (*novelty* sensory cue features in the present study: *jaw dropped*, *eyebrows went up*, *opened his or her eyes widely*). Three of the GRID features, *blushed*, *showed tears* and *changed melody of speech*, were not included in any of the four dimensions as they had loadings less than 0.6 on all of these.

It must be stressed that there are other GRID features that load on each of the four dimensions apart from the features presented above. However, these were not selected in the present study because they did not comprise the sensory cue elements that could be encoded by socially interactive robots. As the GRID questionnaire is suited to provide a precise characterisation of the dimensional values of the various emotions and the statistical differences between the languages, the extent to which each of the dimensions can be represented by a reduced number of sensory cue features is uncertain. The results of the present study should therefore be viewed as initial indications about the overall dimensionality of the respective emotions in English and Polish, which would merit further investigation and confirmation.

## Analyses and Results

Intra-linguistic and inter-linguistic analyses were performed on the GRID data. For both of these, the results are grouped and presented in Tables 1, 2, 3, 4, 5 and 6 on the basis of emotions in the following order: sadness, joy, fear and anger. It was decided that anger should be presented last as it is potentially the least equivalent between Polish and British English on account of the two distinct Polish concepts, *złość* and *gniew*.

**Table 1:** Means and statistics for *Sadness*, *Joy*, *Fear* and *Anger* on selected behavioural expression and body activity GRID features

Feature	Means				
	*Sadness*	*Joy*	*Fear*	*Anger*	*F* (3,129), *p*
Physiological features					
Felt shivers	5.25^F^	5.45^F^	7.94^S;J;A^	4.88^F^	13.97, < 0.001
Heartbeat slowing down	5.31^J;F;A^	3.09^S^	2.72^S^	2.09^S^	15.39, < 0.001
Heartbeat getting faster	5.28^J;F;A^	7.45^S^	8.58^S^	8.06^S^	21.25, < 0.001
Breathing slowing down	5.13^F;A^	4.15^F;A^	2.44^S;J^	2.22^S;J^	18.57, < 0.001
Breathing getting faster	5.41^F;A^	6.48^F;A^	8.19^S;J^	7.72^S;J^	16.32, < 0.001
Perspired/moist hands	4.72^F;A^	5.18^F;A^	8.08^S;J^	7.31^S;J^	30.4, < 0.001
Sweat	4.16^F;A^	4.76^F;A^	7.97^S;J^	7.06^S;J^	33.02, < 0.001
Felt hot	4.53^F;A^	5.58^A^	5.92^S;A^	7.84^S;J;F^	13.94, < 0.001
Blushed	3.94^J;A^	6.58^S;F^	4.33^J;A^	6.16^S;F^	14.94, < 0.001
Felt cold	6.09^J;A^	2.39^S;F^	7.03^J;A^	3.66^S;F^	39.45, < 0.001
Facial expression features					
Smiled	2.59^J^	8.61^S;F;A^	2.17^J^	2.06^J^	111.98, < 0.001
Jaw dropped	4.53^J^	6.42^S^	5.53	5.56	3.56, < 0.05
Pressed lips together	5.41^J;A^	2.91^S;F;A^	6.03^J^	7.34^J;S^	31.05, < 0.001
Eyebrows went up	3.97^J;F;A^	6.33^S^	6.56^S^	5.59^S^	8.71, < 0.001
Frowned	7.34^J;F^	2.09^S;F;A^	5.89^J;A^	7.59^J;F^	70.59, < 0.001
Closed his or her eyes	6.94^J^	4.39^S;F^	6.72^J^	5.56	9.27, < 0.001
Opened his or her eyes widely	4.25^J;F;A^	7.61^S^	7.64^S^	6.84^S^	25.06, < 0.001
Showed tears	8.16^J;A^	6.33^S^	7.03	6.84^S^	5.6, < 0.01
Body movement features					
Abrupt bodily movements	4.69^J;F;A^	6.33^S^	7.00^S^	7.41^S^	11.48, < 0.001
Moved towards people or things	4.69^J;A^	6.33^S;F^	7.00^J^	7.41^S^	7.18, < 0.001
Withdrew from people or things	6.16^J^	2.70^S;F;A^	6.00^J^	4.91^J^	18.76, < 0.001
Paralinguistic features					
Increased the volume of voice	4.34^J;F;A^	7.30^S^	6.67^S;A^	7.94^S;F^	21.36, < 0.001
Decreased the volume of voice	6.63^J;F;A^	2.94^S;F^	4.89^S;J^	4.06^S^	16.18, < 0.001
Had a trembling voice	7.72^J^	5.45^S;F;A^	8.03^J^	6.94^J^	13.39, < 0.001
Had an assertive voice	3.78^J;F;A^	5.88^S;A^	5.19^S;A^	7.63^S;J;F^	17.95, < 0.001
Changed melody of speech	6.59	7.45	7.03	7.22	1.28, n. s.
Produced speech disturbances	6.84^J^	5.42^S;F;A^	7.19^J^	7.09^J^	5.49, < 0.01
Spoke faster	4.44^J;F;A^	7.03^S^	6.69^S^	7.09^S^	11.74, < 0.001
Spoke slower	7.06^J;F;A^	3.76^S^	4.58^S^	3.97^S^	17.04, < 0.001
Linguistic features					
Fell silent	7.66^A;J^	3.15^A;F;S^	7.14^J;A^	4.84^S;J;F^	36.8, < 0.001
Produced a short utterance	6.06^J^	4.64^S;F;A^	6.72^J^	6.81^J^	8.28, < 0.001
Produced a long utterance	4.66^J;A^	6.12^S^	5.31	6.34^S^	4.9, < 0.01
Other					
Wanted to sing and dance	2.88^J;A^	8.09^S;F;A^	2.00^J^	1.69^S;J^	94.14, < 0.001

Significant differences between the means are denoted by the first letters of the emotions: ^S^ *sadness*, ^J^ *joy*, ^F^ *fear* and ^A^ *anger*

**Table 2:** Means and statistics for *Smutek*, *Radość*, *Strach*, *Złość* and *Gniew* on selected behavioural expression and body activity GRID Features

Feature	Means					
	*Smutek*	*Radość*	*Strach*	*Złość*	*Gniew*	*F* (4,124), *p*
Physiological features						
Felt shivers	4.24^St^	5.15^St^	7.19^Sm;R;Z;G^	5.26^St^	5.5^St^	5.75, <0.001
Heartbeat slowing down	6.52^R;St;Z;G^	3.00^Sm^	2.94^Sm^	2.21^Sm^	2.70^Sm^	11.3, <0.001
Heartbeat getting faster	3.52^R;St;Z;G^	7.30^Sm^	8.50^Sm^	7.89^Sm^	7.83^Sm^	24.49, <0.001
Breathing slowing down	6.33^R;St;Z;G^	3.44^Sm;Z^	2.66^Sm^	1.32^Sm;R^	2.93^Sm^	14.33, <0.001
Breathing getting faster	3.10^R;St;Z;G^	6.52^Sm;St;Z;G^	8.31^Sm;R;^	8.68^Sm;R^	7.97^Sm;R^	47.77, <0.001
Perspired/moist hands	3.24^St;Z;G^	4.81^St;G^	7.75^Sm;R^	6.00^Sm^	6.97^Sm;R^	16.07, <0.001
Sweat	2.67^St;Z;G^	3.89^St;Z;G^	7.84^Sm;R^	6.11^Sm;R^	7.20^Sm;R^	23.29, <0.001
Felt hot	2.33^R;St;Z;G^	5.70^Sm;Z;G^	6.91^Sm^	8.37^Sm;R^	7.87^Sm;R^	31.59, <0.001
Blushed	2.24^R;Z;G^	6.78^SmSt^	3.22^R;Z;G^	6.21^Sm;St^	6.50^Sm;St^	21.61, <0.001
Felt cold	6.67^R;Z^	2.15^Sm;St;G^	6.16^R;Z^	3.00^Sm;St^	4.83^R^	14.11, <0.001
Facial expression features						
Smiled	1.81^R^	8.52^Sm;St;Z;G^	1.47^R^	1.58^R^	1.70^R^	126.49, <0.001
Jaw dropped	3.86	3.81	3.22	3.26	3.47	0.34, n. s.
Pressed lips together	6.00^R;Z^	2.30^Sm;St;Z.G^	5.47^R;Z;G^	8.47^Sm;R;St^	7.40^R;St^	24.1, <0.001
Eyebrows went up	2.43^R;Z;G^	4.74^Sm^	4.41	5.11^Sm^	4.97^Sm^	3.87, <0.001
Frowned	4.38^R;Z;G^	2.41^Sm;Z.;G^	3.78^Z;G^	8.58^Sm;R;St^	7.07^Sm;R;St^	34.31, <0.001
Closed his or her eyes	7.67^R;Z;G^	4.63^Sm^	6.47	4.47^Sm^	5.47^Sm^	5.61, <0.001
Opened his or her eyes widely	2.10^R;St;G^	6.85^Sm;Z^	7.09^Sm;Z^	3.63^R;St;G^	5.77^Sm;Z^	23.45, <0.001
Showed tears	8.71^R;St;G^	6.89^Sm^	5.91^Sm^	7.00	6.33^Sm^	5.44, <0.001
Body movement features						
Abrupt bodily movements	2.81^R;St;Z;G^	6.56^Sm^	6.22^Sm;Z;G^	7.95^Sm;St^	8.07^Sm;St^	22.28, <0.001
Moved towards people or things	2.86^R^	6.81^Sm;St;G^	4.50^R^	5.00	4.80^R^	7.2, <0.001
Withdrew from people or things	7.10^R;Z^	1.96^Sm;St;Z;G^	6.34^R;Z^	4.37^Sm;R;St^	5.53^R^	19.0, <0.001
Paralinguistic features						
Increased the volume of voice	2.00^R;St;Z;G^	7.74^Sm;St^	4.84^Sm;R;Z;G^	8.05^Sm;St^	8.43^Sm;St^	50.08, <0.001
Decreased the volume of voice	6.81^R;St;Z;G^	3.37^Sm;Z^	2.88^Sm^	1.42^Sm;R^	2.20^Sm^	22.32, <0.001
Had a trembling voice	8.05^R^	5.63^Sm;St^	7.97^R^	6.16	6.53	5.69, <0.001
Had an assertive voice	1.90^R;Z;G^	5.07^Sm;St;Z;G^	3.06^R;Z;G^	7.32^Sm;R;St^	7.97^Sm;R;St^	45.64, <0.001
Changed melody of speech	7.81	7.19	7.56	8.42	8.07	2.01, n. s.
Produced speech disturbances	7.52	6.15	7.56	6.79	7.13	2.13, n. s.
Spoke faster	2.90^R;St;Z;G^	7.41^Sm^	6.00^Sm;Z;G^	8.16^Sm;St^	7.67^Sm;St^	23.55, <0.001
Spoke slower	7.33^R;St;Z;G^	2.70^Sm;St^	4.97^Sm;R;Z^	1.79^Sm;St;G^	3.67^Sm;Z^	22.29, <0.001
Linguistic features						
Fell silent	8.24^R;Z;G^	2.81^Sm;St;G^	6.59^R;Z^	4.16^Sm;St^	5.97^Sm;R^	17.0, <0.001
Produced a short utterance	7.24^R;St^	3.48^Sm;G^	5.03^Sm^	5.16	6.10^R^	8.38, <0.001
Produced a long utterance	3.95^R;G^	6.07^Sm;St^	3.34^R;Z;G^	5.79^St^	6.27^Sm;St^	9.6, <0.001
Other						
Wanted to sing and dance	1.19^R^	8.15^Sm;St;Z;G^	1.69^R^	1.32^R^	2.37^R^	84.34, <0.001

Significant differences between the means are denoted by the first letters of the emotions: ^Sm^ *smutek*, ^R^ *radość*, ^St^ *strach*, ^Z^ *złość* and ^G^ *gniew*

**Table 3:** Means and *t* test results for *sadness* versus *smutek*

Feature	British English mean	Polish mean	*t*	*df*	*p*
Heartbeat getting faster	5.28	3.52	−2.66	51	<0.01
Breathing getting faster	5.41	3.1	−4.02	51	<0.01
Perspired/moist hands	4.72	3.24	−2.72	51	<0.01
Felt hot	4.53	2.33	−4.07	51	<0.01
Blushed	3.94	2.24	−3.14	51	<0.01
Frowned	7.34	4.38	−4.68	27.91	<0.01
Opened his or her eyes widely	4.25	2.1	−4.7	48.93	<0.01
Abrupt bodily movements	4.69	2.81	−3.02	51	<0.01
Moved towards people or things	5.47	2.86	−4.02	51	<0.01
Increased the volume of voice	4.34	2.0	−4.6	49.66	<0.01
Had an assertive voice	3.78	1.9	−4.34	50.96	<0.01
Wanted to sing and dance	2.88	1.19	−3.66	34.79	<0.01

**Table 4:** Means and *t* test results for *joy* versus *radość*

Feature	British English mean	Polish mean	*t*	*df*	*p*
Jaw dropped	6.42	3.81	−4.2	58	<0.01

**Table 5:** Means and *t* test results for *fear* versus *strach*

Feature	British English mean	Polish mean	*t*	*df*	*p*
Jaw dropped	5.53	3.22	−3.69	66	<0.01
Eyebrows went up	6.56	4.41	−3.67	58.52	<0.01
Frowned	5.89	3.78	−3.72	57.68	<0.01
Increased the volume of voice	6.67	4.84	−2.87	55.06	<0.01
Decreased the volume of voice	4.89	2.88	−3.52	66	<0.01
Had an assertive voice	5.19	3.06	−3.87	66	<0.01
Produced a short utterance	6.72	5.03	−3.12	49.42	<0.01
Produced a long utterance	5.31	3.34	−3.67	66	<0.01

**Table 6:** Means and *t* test results for *anger* versus *złość* and *anger* versus *gniew*

Feature	Anger mean	Złość mean	Gniew mean	*t*	*df*	*p*
Breathing slowing down	2.22	1.32		−2.81	47.36	<0.01
Breathing getting faster	7.72	8.68		2.98	38.4	<0.01
Jaw dropped	5.56^ab^	3.26^a^	3.47^b^	−3.44^a^	49^a^	<0.01^a^
				−3.5^b^	60^b^	<0.01^b^
Pressed lips together	7.34	8.47		2.73	49	<0.01
Frowned	7.59	8.58		3.04	47.92	<0.01
Opened his or her eyes widely	6.84	3.63		−4.25	28.86	<0.01
Moved towards people or things	7.16^ab^	5.0^a^	4.8^b^	3.07^a^	24.34^a^	<0.01^a^
				−4.2^b^	45.23^b^	<0.01^b^
Decreased the volume of voice	4.06^ab^	1.42^a^	2.2^b^	−5.53^a^	44.64^a^	<0.01^a^
				−3.39^b^	59.16^b^	<0.01^b^
Changed melody of speech	7.22	8.42		2.91	48.36	<0.01
Spoke slower	3.97	1.79		−4.36	48.86	<0.01

^a^ means and *t* test results for *anger* versus *złość*

^b^ means and *t* test results for *anger* versus *gniew*

### Intra-Linguistic Analyses

Two separate MANOVAs were performed on the British English and Polish GRID samples. For both British English and Polish, the dependent variables were the thirty-three behavioural expression/body activity features (see Tables 1 and 2). The independent variable for British English was *emotion* (*sadness*, *joy*, *fear* and *anger*), with the dependent variable for Polish also being *emotion* (*smutek*, *radość*, *strach*, *złość* and *gniew*). There was a significant interaction between *emotion* and behavioural expression/body activity features for both British English (*F* (33, 99) = 8.83, *p* < 0.01) and Polish (*F* (33, 132) = 7.14, *p* < 0.01), showing that the differences between the emotions were therefore dependent on the behavioural expression/body activity features for both languages. The univariate effects for each of the behavioural expression/body activity features are shown in the last column of Table 1 (British English) and Table 2 (Polish). For features that violated homogeneity of variance on Levene’s test of equality of error variance, a more stringent α level was set at 0.01 (cf. [29]). The Tukey HSD post hoc test was performed on the emotions in both British English and Polish. Significant differences between the British English emotions (Table 1) are shown by superscripted letters to the right of the means that denote each of the emotions as follows: sadness = ^S^, joy = ^J^, fear = ^F^ and anger = ^A^; and similarly for the Polish means (Table 2) as follows: smutek = ^Sm^, radość = ^R^, strach = ^St^, złość = ^Z^ and gniew = ^G^. For example, in Table 1, the *felt shivers* feature shows 5.25^F^ in the column for *sadness* column, 5.45^F^ in the column for *joy*, 7.94^S;J;A^ in the column for *fear* and 4.88^F^ in the column for *anger*. This means that *felt shivers* is significantly more likely to occur for *fear* than *sadness*, *joy* and *anger*.

### Inter-Linguistic Analyses

Five independent *t* tests were performed between the equivalent emotions in British English and Polish (*sadness*—*smutek*, *joy*—*radość*, *fear*—*strach*, *anger*—*złość* and *anger*—*gniew*). To ensure that the cumulative Type 1 error was below 0.05, the Bonferroni correction was applied, resulting in an α level of 0.01. Tables 3, 4, 5 and 6 show the means for the behavioural expression/body activity features where there were significant differences between British English and Polish.

The results of these intra- and inter-linguistic analyses are presented for the Polish and British English equivalents of each of the emotions in separate sections below.

### Sadness (British English *Sadness* and Polish *Smutek*)

#### GRID Results

Tables 1 and 2 show that relative to the other emotions both British English *sadness* and Polish *smutek* are characterised by lower *arousal* (e.g. slower heartbeat and breathing, and less sweating). However, the significantly lower values for *heartbeat getting faster*, *breathing getting faster* and *felt hot* presented in Table 3 for *smutek* suggest that although this emotion is less activated in both languages, it is more so in Polish. As expected, *power* is also lower in both of these emotions, as evidenced by the linguistic and paralinguistic features *decreased the volume of voice*, *fell silent* and *spoke slower*. *Smutek* is somewhat lower in *power* compared with *sadness* (see *increased the volume of voice* and *had* *an assertive voice*, Table 3), showing that despite *power* being low in both languages, it is to some extent lower in Polish. The negative *valence* that is characteristic of *sadness* and *smutek* is shown by the less likelihood of smiling, relatively more withdrawal from people or things, and feeling cold in Tables 1 and 2. Although negative *valence* characterises both *sadness* and *smutek*, the relative *valence* between these two emotions is less clear, with significantly higher ratings for *sadness* on both the positively valenced features of *moved towards* and *sing and dance* and the negatively valenced feature of *frowned* (Table 3). There is also evidence that *sadness* and *smutek*, relative to the other emotions, are more predictable, as shown by the relatively low scores on *opened his or her eyes widely* and *eyebrows went up* in Tables 1 and 2, with *smutek* having more of an element of predictability than *sadness*, as shown in Table 3 by the lower likelihood of the opening of eyes.

#### Corpus Analysis Results

Language corpus analysis results shed more light on the properties identified in the GRID questionnaire. Frequencies of occurrence of language-specific Part-Of-Speech (POS) patterns involving emotion words provide further information with respect to the properties of culturally bound emotion concepts and their linguistic expression. The corpus data are either fully or partly formalizable in terms of componential and cluster analyses. Frequencies of occurrence of the linguistic realisation of a particular emotion or emotion clusters in the two languages can be juxtaposed to the associated valence, arousal and dominance values of the emotion words, involving aggregates of bodily gestures as well as behavioural and language-related properties of emotions as particular *tertia comparationis* in the present analysis.

A characteristic feature of the English–Polish corpus-based contrasts concerning sadness (see appendices for sadness and smutek Nouns, Adjectives, Verbs) is the more frequent collocate links between *smutek* and Polish adjectives expressing higher intensity (*przejmujący* ‘piercing, bitter’, *bezbrzeżny* ‘infinite lit. unbounded, boundless’, etc.), when compared with English. Similarly, the top verbal collocations include metaphoric phrases showing a higher degree of emotion intensity in Polish than in English (*pogrążyć* ‘plunge’, *ogarnąć* ‘overwhelmed (by sadness)’, *topić* ‘sunk (in sadness)’, *napawać* ‘filled with (sadness)’). Examples of English and Polish concordances identify the characteristic properties of these emotions:

sadness concordances

1. The fear and great sadness are expressed through the cry and the use of shiver
2. tears of real sadness
3. her hands, for once, lying idle in her lap, an expression of infinite sadness on her face
4. let out a terrible call and shriek of sadness

smutek ‘sadness’ concordances

(5) Smutek na twarzy ‘sadness on face’
(6) Anders pokiwał ze smutkiem głową ‘Anders nodded his head with sadness’

In addition to the bodily reactions involving the emotion of sadness, concordances can reveal a range of shades of the emotion, by using relevant modifying phrases such as *great,* *gentle*, *rich*, *full,* *extreme*, *enormous*, *dull, dignified* and *deep*:
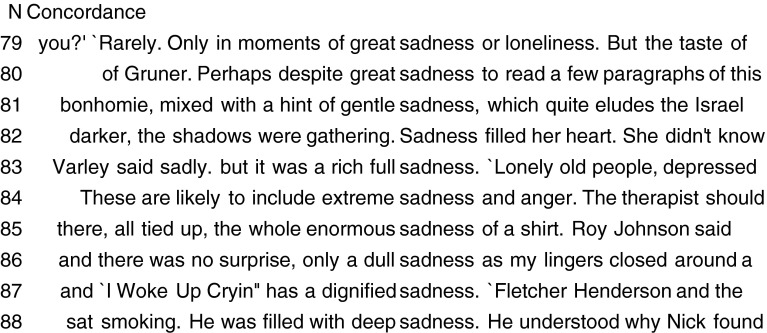


#### GRID Versus Corpus Results

The cross-cultural consistency shown between the GRID and corpora results on the power dimension suggests that this is a salient feature distinguishing between Polish and British English sadness. The GRID results showing an element of lower *power* in *smutek* compared with *sadness* (*increased the volume of voice* and *had* *an assertive voice*) are also reflected in the higher degree of emotion intensity in Polish metaphoric phrases than in English (*pogrążyć* ‘plunge’, *ogarnąć* ‘overwhelmed (by sadness)’, *topić* ‘sunk (in sadness)’, *napawać* ‘filled with (sadness)’), which clearly point to relatively lower *power* in Polish. Consistent with this, the concordance data for *sadness* refer to the letting out of ‘a terrible call and shriek of sadness’—an action characterising relatively higher *power*. The relatively lower *arousal* and somewhat more predictability in *smutek* compared with *sadness* is partly supported by some corpus data, which generate *przygnębienie* ‘depression’, *melancholia* ‘melancholy’ or *apatia* ‘apathy’ as contextual collocational forms in Polish, while the top most emotion collocate form in English is *anger*.

### Joy (British English *Joy* and Polish *Radość*)

#### GRID Results

In Tables 1 and 2, it can be seen that *joy* and *radość* are similar, positive emotions that invite social engagement (e.g. relatively high values for smiling, moving towards people or things and wanting to sing or dance, but a low score for falling silent). There is only one significant difference between these emotions (*jaw dropped* is higher in *joy*), which shows that *joy* (mean: 6.42) might entail relatively more surprise (Table 4) relative to *radość* (mean: 3.81); however, neither emotion has an extreme rating on this feature.

#### Corpus Analysis Results

The GRID *joy* results are complemented by the corpus data. The immediate context of English *joy*, expressed in terms of verbal collocates, indicates a strong element of bodily reaction engagement (*bring, jump, experience, discover, watch, dance, share, express, ride, weep, behold, burst, fill, leap, sing, shout, kiss*), while a clear feature of *radość* is its more social (collectivist) character (*dawać* ‘give’, *przynosić* ‘bring’, *przyjąć* ‘receive’, *witać* ‘greet’*, dzielić* ‘share’), but also the presence of a certain amount of control (*kontrolować* ‘control’, *chować/ukrywać* ‘hide’). Less frequent collocates of Polish *radość* ‘joy’ also include activities such as in (8); however, their frequencies are all below those in English.

(8) radość ‘joy’ collocates of lower frequencies

śmiech ‘laugh’

skakać ‘jump’

płakać ‘cry’

łza ‘tear’

krzyczeć ‘shout’

tańczyć ‘dance’

śpiewać ‘sing’

klaskać ‘clasp (hands)’

piszczeć ‘squeal’

drżeć ‘tremble’

#### GRID Versus Corpus Results

The corpora results add more in-depth information to the GRID findings on the comparison between *joy* and *radość*. The verbal collocates that characterise bodily reaction engagement (e.g. *dance*, *laugh*, *sing*, *kiss* and *express*) in both *joy* and *radość* are consistent with the social engagement action features shown in the GRID results for these emotions. However, frequencies of these bodily reaction engagement collocates are lower for *radość*, suggesting that *joy* might be characterised by more of an element of outward action and engagement than *radość*. This possibility is consistent with a certain amount of control (*kontrolować* ‘control’, *chować/ukrywać* ‘hide’) in the verbal collocates of *radość*. Verbal collocates also suggest that *radość* has a certain social (collectivistic) element compared with *joy* (e.g. *dawać* ‘give’, *przynosić* ‘bring’, *przyjąć* ‘receive’, *witać* ‘greet’ *and dzielić* ‘share’).

### Fear (British English *Fear* and Polish *Strach*)

#### GRID Results

Tables 1 and 2 show that the clearest defining characteristic of *fear* and *strach* is high *arousal* (e.g. *felt shivers*, *heartbeat getting faster*, *breathing getting faster* and *sweat*). However, there are no significant differences between these two emotions on this dimension. On the whole, *fear* and *strach* do not present a clear pattern in terms of *novelty*; however, the somewhat higher element of surprise in *fear*, relative to *strach*, can be seen in *jaw dropped* and *eyebrows went up*, which are relatively higher in *fear*. The overall pattern shows that *fear* and *strach* are characterised by negative *valence*, as evidenced by a lack of smiling, a trembling voice and not wanting to sing and dance (Tables 1, 2). However, the relative *valence* of these two emotions is not clear as Table 5 shows that *fear* has a relatively more negative *valence* on the features *frowned* and produced a *short utterance*, but *strach* has a more negative *valence* than *fear* on *produced a long utterance*. A comparison between these two emotions on the *power* dimension is also not clear as *fear* is relatively more likely to have a loud, assertive voice, but to also have a quieter voice than *strach*.

#### Corpus Analysis Results

Detailed semantic analysis of the meaning of corpus-based individual collocates in both languages was performed manually to identify additional bodily cues of particular emotions and to elaborate on them, e.g. the Noun collocates of Polish fear ‘*strach*’ all specify bodily reactions of Experiencer such as *ze strachu ściśniętym gardłem* (lit. ‘with a throat squeezed/pressed with fear’), i.e. to have a lump in one’s throat. The collocates also describe fear as experienced with *zaciśnięte ze strachu oczy* ‘eyes closed (lit. pressed) with fear’ or the opposite *rozszerzone strachem oczy* ‘eyes widened with fear’, as evident in the corpus data. The corpus data provide detailed support and extension of the GRID features and identify features, which would be considered opposite to each other when taken verbatim (eyes opened or eyes closed). However, it can be reasoned that these are justified if different fear scenarios are taken into consideration (see Lewandowska-Tomaszczyk and Wilson [18] for a discussion on different *fear* scenarios). Descriptions of face and facial gestures also provide more detail: *zastygła w strachu twarz* ‘face solidified in fear’, *ściągnięta strachem twarz* ‘face puckered with fear’*, skrzywiona ze strachu twarz* lit. ‘face bent/twisted/grimaced with fear’. The GRID *fear*—*strach* results are consistent with the collocates of the fear words in both languages. Our previous analysis of fear in English and Polish [18] revealed two distinct fear scenarios: one in which fear paralyses, more frequent in Polish than English, and another where fear is controlled or conquered, more frequent in English than Polish. These results are coherent with the verbal collocates of *fear* and *strach* reported in the appendices (e.g. English [*fear* fight scenario]: *overcome*, *confirm*, *raise*, *lose*, *dismiss*, *dispel*, *ease* and *conquer*; Polish: *budzić* ‘wake (metaphoric)’, *żyć* ‘live’, [*fear* fright scenario] *paść* ‘fall down’, *trząść/drżeć* ‘tremble/shake’, *ogarniać* ‘overwhelm’, *umierać* ‘die’, *paraliżować* ‘paralyse’, *napędzić* ‘urge/cause’, *najeść* lit. ‘(be) eaten up’ and *oblecieć* ‘(be) overwhelmed’).^3^

Lists of concordances provide materials concerning the Sources (Stimuli) of fear as in the list below (*fear of getting lost*, *fear of God,* etc.):

(9) fear concordances

 
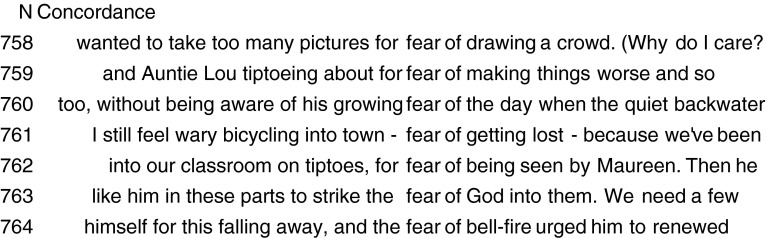


#### GRID Versus Corpus Results

The noun collocates in the Polish corpus data describe fear as experienced with *rozszerzone strachem oczy* ‘eyes widened with fear’. This suggests an element of surprise in *strach*, which is inconsistent with the GRID results that show a somewhat higher element of surprise in *fear* in comparison with *strach* (higher values for *fear* on *jaw dropped* and *eyebrows went up*). The verbal collocates in the corpora data are consistent with the *fight*—*fright* distinction that Lewandowska-Tomaszczyk and Wilson [18] observed for British English and Polish, respectively. Whereas the British English collocates such as *overcome*, *dismiss*, *dispel* and *conquer* suggest an energising, higher degree of power that might overcome one’s fear, the Polish collocates (e.g. *paść* ‘fall down’, *trząść/drżeć* ‘tremble/shake’, *ogarniać* ‘overwhelm’, *umierać* ‘die’, *paraliżować* ‘paralyse’) point to a less powerful response to fear in which the individual more passively submits to fear. The Polish–British English comparison on the *power* dimension in the GRID results was not consistent with this pattern.^4^

### Anger (British English *Anger* and Polish *Złość* and *Gniew*)

#### GRID Results

It is important to assess British *anger* vis-à-vis the two forms of Polish anger, *złość* and *gniew*. Tables 1 and 2 clearly show that these three types of anger are high on *arousal*, as exemplified by faster heartbeat and breathing, and feeling hot. The faster breathing of *złość* in comparison with *anger* suggests that *złość* has somewhat more of an element of relatively higher *arousal* (see features *breathing slowing down* and *breathing getting faster*, Table 6). The features pertaining to novelty (*jaw dropped*, *eyebrows went up*, and *opened his or her eyes widely*) do not present a clear pattern for the three types of anger. However, despite none of these emotions being particularly associated with *surprise*, Table 6 shows that the dropping of the jaw is significantly more likely to occur in *anger* than in both *złość* and *gniew* and the opening of the eyes is more characteristic of *anger* than *złość*. *Anger*, *złość* and *gniew* are characterised by relatively more negative *valence* (e.g. lower values on *smiled*, but higher values on *frowned* and *pressed lips together*). When comparing the three emotions on *valence*, it is evident that *złość* has a relatively more negative *valence* in comparison with *anger* (significant differences on three features in Table 6: *złość* has higher values for *pressed lips together* and *frowned*, but a lower value for *moved towards people or things* than *anger*) than *gniew does* (a significant difference on only one feature in Table 6: *gniew* has a lower value than anger for *moved towards people or things*). Tables 1 and 2 show that anger is characterised by relatively high *power* (e.g. *increased volume of voice*, *had an* *assertive voice*, and *spoke faster*), with *złość* being somewhat relatively higher in *power* than *anger* (see lower values for *złość* on *decreased volume of voice* and *spoke slower* in Table 6).

#### Corpus Analysis Results

The differences in distinct manifestations of *anger* are captured in Polish in terms of two distinct EESs, one *gniew* ‘anger 1’ and the other *złość* ‘anger 2’. *Gniew* is more controllable, and is therefore easier to suppress (note *tłumić* ‘suppress’ Verbs in the collocate table) when compared with *złość* (no *suppress* Verbs), and *gniew* also has a more definite reason (compare Adjectives for złość *bezsilny* ‘powerless‘, *bezrozumny* ‘unreasonable, irrational, unjustified’ with gniew *słuszny* ‘right, rational, justified’).

*Gniew* typically occurs when an Experiencer reacts to another person (stimulus) who directs bodily or verbal acts towards the Experiencer and is considered to be an Attacker by the Experiencer. This emotion is partly controllable and is connected with some form of reaction from the Experiencer, who aims to stop the Attacker’s action. The Experiencer feels hostility or detachment as a consequence of what the Attacker did to him/her.

*Złość* can accompany *gniew* but it can also be a reaction to negative or unfavourable conditions or circumstances (Stimuli), and it is therefore a reaction of displeasure towards something and can also be related to a feeling of antagonism towards someone or irritation towards something.

Both the less frequent collocates as well as the concordances of English a*nger* and Polish *gniew* ‘anger 1’ and *złość* ‘anger 2’ uncover contextual characteristics of each of the emotions.

**anger** **concordances**

(10) His voice shook with anger
(11) Voice trembling with anger
(12) Shaking with anger
(13) His eyes flashed with anger
(14) A blaze of anger flashed across his face
(15) They both splutter with anger
(16) Emily felt anger run through her

**gniew** **‘anger 1’ concordances**

(17) ze ściśniętą z gniewu twarzą ‘with face squeezed (tight) with anger’
(18) zadręczoną gniewem twarzą ‘face tormented with anger’
(19) w gniewie twarz zakrzepłą ‘face stiffened with anger’
(20) rozdygotanym od gniewu głosem ‘voice shivering with anger’
(21) kipiącym gniewem głosem ‘voice boiling with anger’
(22) zduszonym/stłumionym od gniewu głosem—‘voice muffled/quashed/dampened with anger’

**gniew** **‘anger 1’ collocates Verbs** (lower frequencies)

(23) kopnąć ‘kick’
(24) krzywić ‘twist’
(25) cisnąć ‘throw (things at sth)’

**złość** **‘anger 2’ concordance**

(26) Wykrzywione złością twarze, przerażone oczy ‘face grimaced (twisted, frowned) with anger, terrified eyes’

**złość** **‘anger 2’ collocates Verbs** (lower frequencies)

(27) uderzyć ‘hit’
(28) tupać ‘stamp/thump one’s feet’
(29) krzyczeć ‘shout’
(30) syknąć ‘hiss’
(31) płakać ‘cry/weep’
(32) szarpnąć ‘tear’
(33) prychnąć ‘snart’
(34) ciskać ‘throw (things) at smth’

#### GRID Versus Corpora Results

The collocation and concordance results for *anger*, *złość* and *gniew* are consistent with the GRID results as they show that these anger emotions are high on *arousal* and *power* and have a relatively more negative *valence*. However, although the collocation and concordance results, unlike the GRID results, do not provide a clear pattern regarding how *anger*, *złość* and *gniew* differ on these dimensions, they give additional information to what the GRID findings show. Further studies are necessary to uncover, for example, the possible sensory cues associated with the more controllable, easier to suppress *gniew* (*tłumić* ‘suppress’) in its comparison with *złość*.

## Discussion

GRID and corpus linguistic analyses performed on the selected expressive behavioural and body activity features produced profiles that one would generally expect for the basic emotions joy, sadness, fear and anger and that are consistent with those observed by Scherer and Walbott [27]. The analyses also produced interesting cross-linguistic differences between Polish and British English for each of the emotions tested.

The corpus data provide support for and enrich the GRID findings, both with reference to body movements, gestures and activities resulting from a particular emotion. The points of reference for the comparison between the GRID questionnaire results and the corpus analysis are respective body movements, gestures and activities performed by an Experiencer, usually expressed as Verbs or Verbal forms, and the emotion description and evaluation as verbalised in terms of Adjectives in the relevant linguistic materials.

### Intra-Linguistic Differences

The GRID results regarding the differences between sadness, joy, fear and anger showed that there were similarities in the general contrastive pattern of these emotions within British English and within Polish. The corpus data provide additional information on emotion properties and their recognition from both intra- as well as inter-linguistic perspectives, particularly in the form of collocates with which the emotion terms are most frequently used (see "Appendix 1"). The most informative are the Verbal and Adjectival collocates, while the Noun collocations are less frequent, with a lower significance level. In two cases (sadness and joy), nominal collocations cannot be generated due to the frequencies being below the threshold level of 5.

The pattern that emerged from the GRID analyses comparing British English *sadness* and the other British English emotions was similar to the one between Polish *smutek* and the rest of the Polish emotions. Both *sadness* and *smutek* are characterised by relatively lower *arousal* (e.g. slower heartbeat and breathing, and less sweating), lower *power* (e.g. *decreased the volume of voice*, *fell silent* and *spoke slower*), more negative *valence* (e.g. *withdrawal from people or things*, *felt cold* and less likelihood of smiling) and higher predictability (as evidenced by lower *novelty*—e.g. *opened his or her eyes widely* and *eyebrows went up*) than the other emotions.

The examined corpora provide additional data on sadness. English *sadness* corpus-generated collocations present a more moderate emotion level with the Verbal collocates *feel*, *tinge and express*. While *sadness* collocates most frequently with the Adjectives *great* and *deep* in our data, the Adjectival collocates of more positive emotions, like joy, are *sheer*, *full* and *pure*. *Deep* is particularly frequent with more negative emotions, as it is metaphorically linked with a lower, lying or weakened position, generally expressed as *feeling down.* Polish *smutek* ‘sadness’ collocates with the Adjectives of high intensity such as great, piercing, and boundless and (frequently metaphoric) Verbs of similarly negative charge (Polish equivalents to plunge/sink/drown).

Joy is generally regarded to be the opposite to sadness, and this was confirmed in the GRID results, which showed that both *joy* and *radość*, in comparison with sadness, fear and anger, are positive emotions that engender social engagement (e.g. relatively high values for smiling, moving towards people or things and wanting to sing or dance). Collocates of English *joy* confirm a high degree of arousal and a positive, energetic reaction to a Stimulus (*jump*, *discover, dance*, etc.). Polish *radość* ‘joy’ can be perceived in corpus data as having a more social character (first two topmost Verbal collocates are two forms of s*prawiać* ‘give/cause (joy)’, the third being—*dawać* ‘give’). It is also indicative of an Experiencer/Current Speaker’s emotion, e.g. the form *mój* ‘my/mine’ is one of the most frequently used collocates.

Similarities were also present in the comparison between *fear* vis-à-vis the other British English emotions and *strach* in relation to the other Polish emotions. These intra-linguistic comparisons showed that the most salient characteristic of both *fear* and *strach* was high *arousal* (e.g. *heartbeat getting faster*, *breathing getting faster*, *felt shivers* and *sweat*). These emotions were also characterised by negative *valence* (e.g. lack of smiling, trembling voice and not wanting to sing or dance). However, a clear pattern did not emerge for either *fear* and *strach* when they were compared with the other emotions on the *novelty* and *power* dimensions.

The relevant corpus materials are revealing in providing more details of the fear event, which turns out to belong to two distinct *fear* scenarios. The two scenarios identified are—a prevailing (top frequencies of collocations) one for English—a fight scenario, signalled by the verbs *allay, overcome, dismiss*, *dispel* and *ease* and the second—less frequent in English—involving a fright scenario and expressed by *raise*, *tremble*, *grow*, etc. Polish *strach* ‘fear’ on the other hand is dominated by the highest frequencies of the forms indicating fear-paralysing effects, causing trembling, shivering, etc., and presents a higher preference for the fright scenario in the corpus data.

The GRID results show that, relative to the other emotions, *anger*, *złość* and *gniew* are all generally characterised by a pattern of relatively high *arousal* (e.g. faster heartbeat and breathing, and feeling hot), negative *valence* (e.g. relatively lower values on *smiled*, but higher values on *frowned* and *pressed lips together*) and high *power* (e.g. *increased volume of voice*, *had an* *assertive voice* and *spoke faster*). These results complement other similar findings (e.g. Scherer and Walbott [27]).

A striking result reported in the English anger collocation tables in "Appendix 1" is the feature of the Adjectival ‘suppress’ and Verbal meanings involving acts of venting, rising, provoking and controlling anger, which make them similar to *fear* on the dimension of control, with widespread eyes, sullen face/mood, emotions growing or suppressed, boiling or vented being, frequently metaphorically expressed, signs of English anger, which are parallel to and elaborate on the GRID features. Metaphor is clearly absent in the GRID questionnaire; however, it adds pertinent characteristic properties to the description of particular emotions.

Differences between Polish *gniew* ‘anger 1’ and *złość* ‘anger 2’, expressed in terms of top frequency properties related to ‘justified anger’ for *gniew* and ‘powerless and unjustified anger’ for *złość*, provide additional evidence both for the intra-linguistic distinction between the two concepts as well as for the inter-linguistic asymmetric relation between English and Polish in this respect. Additionally, it is worth noting that while in the case of joy or fear what can be inferred from the data refers typically to either Sources/Stimuli of emotions or to the Experiencer of emotion who is the current speaker (*moja radość* ‘my joy’; *bojaźń bożą/Boga* ‘God’s/godly fear’), in the case of *gniew* the Nominal and Adjectival collocates point usually to external Experiencers of emotions as in *gniew boży* ‘God’s/godly anger’.

As a whole, this background of knowledge can form a database of features that can be employed in the robotics modelling of encoding and decoding expressive sensory cues pertaining to facial expressions (e.g. Wimmer et al. [38]), motion cues (e.g. Castellano et al. [4]), vocal cues (e.g. Vogt et al. [30]) and physiological measures (e.g. Barber et al. [2]).

### Inter-Linguistic Differences

Differences emerged between British English and Polish for all of the emotions. The GRID results showed that *smutek* has lower *arousal* (e.g. slower heartbeat and breathing, and a lower score for feeling hot), somewhat lower *power* (see relative means of *increased volume of voice* and *had an assertive voice*) and an element of more predictability (less likelihood of the opening of eyes) than *sadness*. The consistency between lower *power* in the GRID results and the lower *power* in Polish corpora metaphoric phrases for *smutek* suggests that this is a salient feature distinguishing between *smutek* and *sadness*.

The only significant difference between *joy* and *radość* in the GRID results showed that the dropping of the jaw is more likely to occur in *joy*, suggesting that this emotion comprises a somewhat relatively greater element of surprise, although neither emotion has an extreme rating on this feature. This is consistent with our most recent observations from our laboratory, showing that whereas surprise is conceptually closer to the *happiness/joy* cluster in British English, it has a more negative *valence* in Polish. Complementing the GRID results, the corpora results further suggest that *joy* is relatively more characterised by more outward action and engagement, less control and less of a social element than *radość*.

The only clear difference between *fear* and *strach* in the GRID results was on the novelty dimension, which showed a greater element of surprise in *fear* than *strach* (e.g. *jaw dropped* and *eyebrows went up*). However, the relatively higher element of surprise in *strach* as shown in *rozszerzone strachem oczy* ‘eyes widened with fear’ in the noun collocates in the Polish corpus data is inconsistent with this. The relative *valence* of *fear* and *strach* is not clear as *fear* has a relatively more negative *valence* thanks to the features *frowned* and *produced a* *short utterance*, but a more positive *valence* than *strach* as suggested by higher ratings in the feature *produced a long utterance*. Similar inconsistencies were seen on the *power* dimension, with *fear* being relatively more likely to have a loud, assertive voice, but to also have a quieter voice than *strach*. The verbal collocates in the corpora data offer a more consistent pattern regarding *power*, showing that whereas *fear* is characterised by power, dominance and control, *strach* is associated with relatively more weakness, submissiveness and passivity.

Turning to anger, the GRID results show that *złość* has somewhat relatively higher *arousal* (higher ratings for *breathing getting faster* and lower ratings for *breathing slowing down*) and higher *power* than *anger* (lower values for *złość* on *decreased volume of voice* and *spoke slower*). In terms of *novelty*, there is evidence that there is more of an element of surprise in *anger* than both *złość* and *gniew*, as signified by the greater likelihood of jaw dropping in *anger* compared with *złość* and *gniew*, and a more likely opening of the eyes in *anger* than *złość*. *Złość* has a relatively more negative *valence* in comparison with *anger* (higher values for *pressed lips together* and *frowned*, but a lower value for *moved towards people or things* in Table 6) than *gniew does* (a significant difference on only one feature: *gniew* has a lower value than *anger* for *moved towards people or things*). On the whole, on the basis of the GRID results, it appears that there are more differences between *anger* and *złość* than between *anger* and *gniew* on expressive sensory features. The corpora results do not offer support for these results pertaining to the emotion dimensions; however, they provide complementary information on the differences between Polish *gniew a*nd *złość*. More specifically, *gniew* is more controllable and easier to suppress and has more of a definite reason than *złość*.

#### Corpus-Based Inter-Linguistic Emotion Profiles

In the case of *sadness* and *joy*, the corpus data provide confirming evidence to the GRID results and enrich details of relevant parameters of EESs.

The GRID *fear*—*strach* results are consistent with the semantic and collocational properties of the corpus data (see appendices for fear and strach Nouns, Adjectives and Verbs), which offer further evidence in support of our previous analysis of fear in English and Polish [18], in which distinct fear scenarios were identified with a more frequent set of *fear-paralysing* expressions in Polish and more frequent elements showing *fear*-*control* and *fear*-*conquering* in English. The present materials point clearly to such cross-cultural differences in the linguistic data—first, English *Verbal collocates* include *allay, overcome, lose, dismiss, dispel, ease and conquer,* while Polish *Verbal collocates* are *budzić* ‘wake (metaphoric)’, *żyć* ‘live’, *paść* ‘fall down’, *trząść/drżeć* ‘tremble/shake’, *ogarniać* ‘overwhelm’, *umierać* ‘die’, *paraliżować* ‘paralyse’, *napędzić* ‘urge/cause’, *najeść* lit. ‘(be) eaten up’ and *oblecieć* ‘(be) overwhelmed’. There are no *fear*-*control* Verbs in the top 15 collocates in Polish. More extensive corpus data provide additional materials, which uncover a heterogenous (polysemous, i.e. multi-meaning) character of the conceptual content of *fear*—*strach* and provide more compelling materials for three distinct scenarios in the Experiencer’s fear-induced behavioural and psycho–physical properties in each of them, i.e. *fright*, *fight* and *flight* effects. When contrasted with Polish, we observed a prevailing fear-control pattern in the English corpus data. The analysis of fear-metaphor types presented in Lewandowska-Tomaszczyk and Wilson [18] provides additional evidence for the English preference of *fight* effect EESs in the corpus materials and a more frequent *fright* scenario in the case of Polish.

In the case of anger, two distinct concepts in Polish (*gniew* and *złość*) function as equivalents to English *anger* and display different collocational profiles, associated with each, which are blended in the English anger EES.

Although the results obtained from the GRID materials and from the corpus data are consistent and show a convincing picture of inter- and intra-linguistic differences in English and Polish emotions, a word of caution is needed. The results should be considered ‘tentative cues’ about possible differences in the overall dimensionality of the respective emotions in English and Polish, which would merit further investigation and confirmation.

The GRID and corpus results clearly show differences in emotions between Polish and British English and add weight to growing evidence showing cross-linguistic and cross-cultural differences in these and other languages (e.g. Lewandowska-Tomaszczyk & Wilson [18], Wilson et al. [37], Alonso-Arbiol and van der Vijer [1], Ogarkova et al. [23], and Ishii [12], as outlined above). Our demonstration of differences between Polish and British English in features pertaining to expressive, sensory cues in basic emotions that are commonly present in everyday social interactions are also consistent with recent studies showing cultural specificity regarding facial [13] and vocal [16] expression and should alert those engaged in the modelling of socially interactive robots to the need of taking such cross-linguistic and cross-cultural differences into account if such robots are to gain social competence in diverse cultures.

### Lessons for Emotion-Sensitive Interactive Robots

What are the lessons arising from our study for emotion-sensitive interactive robots? The first point is that there are a number features that could be detected by robots pertaining to expressive behaviours and bodily activities that accompany emotions, which when analysed as a complete profile can serve to distinguish between joy, sadness, fear, anger and other human emotions. The differences that we have found between British English and Polish are a matter of degree rather than major differences involving the presence or absence of features. For example, the observation that *pressed lips* is rated significantly higher for *złość* than for *anger* means that pressing the lips together is more likely to occur in *złość* than in *anger*, and not that *anger* is devoid of this facial display. In a similar way to a Polish individual who has had substantial experience interacting in the British culture has learnt that British anger is less likely to be accompanied by lip pressing, a robot designed to read the emotions in Polish culture must be retuned if it is to be as successful at recognising emotions in British culture. For this to happen, it is clear that further work is necessary to identify the prototypical expressive behaviour and bodily activity profiles of emotions in Poland, Britain and any other culture in which such robots will be used. In cultures where there are larger discrepancies in the cultural displays of emotion, relatively minor tuning of the feature profile might not suffice. For example, Ekman [6] noted that controlled anger in New Guineans is characterised by parted lips, which is the reverse pattern to the usual pressed lips he found for middle-class Americans. Such differences find some resonance in Wierzbicka’s [35] analogy with music: “‘Anger’ is not a simple key in the keyboard; it is a complex culture-specific tune. The repertoire of emotional tunes differs from culture to culture” (p. 10). For emotion-sensitive interactive robots to function effectively within different cultures, it is important to acknowledge the full spectrum of the differences in the cultural repertoires of emotional tunes. These differences range from the strengths of the emotion features that we have seen differ between Polish and British English in the present paper, through major differences in the actual presence or absence of features that are exhibited, for example, in the expression of anger in New Guinea and the USA, to instances in which there is the lack of an emotion that exists in another culture, as can be seen in the lack of corresponding emotions to English *grief* in German, French, Polish or Russian [34].

A final, fundamental lesson to be gained from the results concerns the different manifestations of the same emotion or types of the same emotion. As Barrett [3] states, the expression of anger, for example, can take many forms depending on the circumstances, including a driver shouting and shaking their fist, an employee sitting quietly in a boardroom while listening to unfair criticism from the boss, or a teacher speaking sternly but cordially to a pupil because of their misdemeanour. This might explain the apparent paradox in our results that show that *fear* is relatively more likely to have a loud, assertive voice, but to also have a quieter voice than *strach*. Whereas the louder, more assertive version of fear is consistent with the manifestation of *fight* scenarios, the presence of a quieter voice in fear is typical of *fright* ones. It is clear that context is a major influence on differences in the outward expression of instances of the same emotion. If socially interactive robots are to accurately decode different manifestations of the same emotion, further work is necessary to develop the modelling needed to integrate expressive, sensory cues and contextual cues. The likely culture-dependent nature of this interplay between expressive and sensory cues presents further challenges to our aspirations regarding the competence of robots in the social sphere.

## Conclusions

The different profiles that we observed for joy, sadness, fear and anger in both Polish and British English show that the behavioural expression and body activity GRID features that were selected can reliably distinguish between the outward expression of different emotions, while the corpus-based analysis provides important data on the circumstantial characteristics typical of particular culture-specific Emotion Events scenarios. Weightings of these features can be used in robotic modelling to create robots that can competently respond to human emotions in social settings.

The cognitive corpus linguistics approach provides information on the probabilities of the occurrence of some linguistic patterns of emotional language use based on their frequencies and distributional criteria. It has enriched the GRID analysis by identifying details of the bodily reaction (e.g. Pol. *i czuła strach, i czuła niemoc* ‘and she was feeling fear, and she was feeling weakness’) and provides more thorough information on causes and Experiencers of particular emotions (e.g. Pol. *Ciotka, bezsilna, umierała ze strachu i modliła sie* ‘Aunt, helpless, was dying of fear and praying’). This methodology more closely determines the contextual conditioning of particular emotions and corresponding behavioural correlates.

By enriching and more deeply specifying the description, the method provides sufficient data to model culture-bound emotional behaviour by resorting to the clusters of preferential conditions present in the implementation of a particular Emotions Event scenario in a given cultural and linguistic context. Thus, the combined GRID and corpus methodologies identify more detailed patterns and cross-linguistic and cross-cultural consistency in emotions.

The different profiles that we observe for the emotions described in the present study in both Polish and British English show that the cognitive corpus method successfully extends the identification of emotion display in different cultures for affective robotics purposes.

From our cross-cultural comparison of Polish versus British English, it would appear that if emotion-sensitive interactive robots are to be employed in different cultures, they need to be tuned to the unique profiles of emotion features that are present in these cultures.

## Appendix 1: Corpus-Based English and Polish Collocations

### HASK Collocation Database

A RAW FREQUENCIES of COLLOCATES, i.e. words typically co-occurring with other ones with a frequency greater than chance.

TTEST—*t* test (probability of statistical significance).

MI—Mutual Information of two random variables is a measure of the variables’ mutual dependence.

Note:

1. All the lexical forms in the tables below are given in their basic lemma forms.
2. Polish emotion collocates (e.g. *wywołać* ‘call forth’ (single act), *wywoływać* ‘call forth (frequentative)) are not marked for such aspectual linguistic form, as it is not of immediate relevance for the present study.

Collocation tables (displayed are collocates of top (1–15) frequencies of occurrence in relevant collocational combinations):

**(1) ENGLISH SADNESS**

**Table:** sadness Adjectives

#	Collocate	POS	*A*	TTEST	MI
1	great	AJ %	41.0	5.33843	2.58833721656818
2	deep	AJ %	16.0	3.70009	3.73738426346774
3	post-coital	AJ %	3.0	1.73068	10.3049601319919
4	unutterable	AJ %	3.0	1.7305	10.12438788635
5	sweet	AJ %	3.0	1.44206	2.57840135288688
6	considerable	AJ %	3.0	0.86792	1.00315912081529
7	certain	AJ %	5.0	0.71226	0.553282186304275
8	real	AJ %	4.0	0.24479	0.188356249117633
9	only	AJ %	4.0	0.22874	0.17522634327603
10	personal	AJ %	3.0	0.14983	0.1305337383146
11	old	AJ %	3.0	−3.03943	−1.46195521031649

sadness Nouns

No results for Nouns

**Table:** sadness Verbs

#	Collocate	POS	*A*	TTEST	MI
1	feel	V %	20.0	3.67358	2.48549111345927
2	tinge	V %	11.0	3.31293	9.81007172803188
3	express	V %	11.0	3.09355	3.8941018385125
4	anger	V %	6.0	2.42786	6.8234922437942
5	bring	V %	5.0	1.06246	0.930012083137449
6	leave	V %	6.0	0.92139	0.680741003706379
7	show	V %	5.0	0.61357	0.462745645764912
8	come	V %	9.0	0.01509	0.00727615734478037
9	see	V %	6.0	−2.22615	−0.932682136020077
10	have	V %	22.0	−12.10608	−1.84037264045196
11	be	V %	85.0	−18.36053	−1.58085903608248

**(2) ENGLISH JOY**

**Table:** joy Adjs

#	Collocate	POS	*A*	TTEST	MI
1	great	AJ %	91.0	8.21199	1.45116082559955
2	sheer	AJ %	31.0	5.46154	4.48146935420587
3	full	AJ %	35.0	4.55693	3.15726586910922
4	pure	AJ %	20.0	4.25519	2.10490928709158
5	parliamentary	AJ %	18.0	3.94945	2.08215907439554
6	christian	AJ %	17.0	3.68695	5.81797661356042
7	greatest	AJ %	14.0	3.3454	1.34100585447265
8	holy	AJ %	11.0	3.05711	2.54405389158389
9	surprise	AJ %	10.0	2.86995	4.76644631292034
10	real	AJ %	18.0	2.7058	4.24178432246699
11	inner	AJ %	9.0	2.57068	3.77915736466989
12	overwhelming	AJ %	7.0	2.49813	2.908174422532
13	sudden	AJ %	8.0	2.4421	1.59839737811546
14	delirious	AJ %	6.0	2.4363	1.2034452214265

joy Nouns

No results for Nouns.

**Table:** joy Verbs

#	Collocate	POS	*A*	TTEST	MI
1	bring	V %	45.0	5.11322	2.07238518478562
2	jump	V %	22.0	4.42203	4.12734149430893
3	experience	V %	17.0	3.72569	3.3750160016443
4	discover	V %	17.0	3.49437	2.71319731258343
5	watch	V %	19.0	3.28279	2.01813319331679
6	dance	V %	12.0	3.22497	3.85660829582823
7	share	V %	15.0	3.18227	2.4872866000158
8	express	V %	15.0	3.09412	2.31400891568958
9	ride	V %	11.0	2.93783	3.1302361482513
10	weep	V %	8.0	2.72744	4.80778658684817
11	behold	V %	7.0	2.622	6.79979781966958
12	burst	V %	7.0	2.42307	3.57059291714063
13	fill	V %	10.0	2.33468	1.93396643371412
14	leap	V %	6.0	2.24295	3.56802080905893
15	sing	V %	7.0	2.11207	2.3096195045729

**(3) ENGLISH FEAR**

**Table:** fear Adjs

#	Collocate	POS	*A*	TTEST	MI
1	worst	AJ %	86.0	8.38425	3.38228100815277
2	greatest	AJ %	34.0	4.21557	1.85185415160397
3	well-founded	AJ %	15.0	3.83541	6.68766164915101
4	irrational	AJ %	15.0	3.63503	4.02469663642858
5	widespread	AJ %	22.0	3.4081	1.87096886251407
6	morbid	AJ %	10.0	3.05668	4.90428559002784
7	respectable	AJ %	13.0	2.99449	2.56081974114867
8	deep-seated	AJ %	9.0	2.90921	5.04627371496703
9	grow	AJ %	25.0	2.85348	1.21993070729522
10	real	AJ %	63.0	2.71845	0.60492121814307
11	genuine	AJ %	17.0	2.64852	1.48342247691651
12	illogical	AJ %	7.0	2.48922	4.07911232811223
13	sudden	AJ %	17.0	2.43948	1.2921614385293
14	superstitious	AJ %	6.0	2.32459	4.29358376850781

**Table:** fear Nouns

#	Collocate	POS	*A*	TTEST	MI
1	anxiety	N %	22.0	4.41778	4.10464990159163
2	reason	N %	39.0	4.37086	1.73647388439119
3	police	N %	37.0	4.20271	1.69395520411614
4	expert	N %	19.0	3.68369	2.69055847619429
5	official	N %	19.0	3.58185	2.48788455526085
6	violence	N %	17.0	3.56933	2.8963478928712
7	loss	N %	23.0	3.47778	1.86337567525088
8	environmentalist	N %	12.0	3.38719	5.4931218444191
9	critic	N %	14.0	3.32956	3.18263204014131
10	death	N %	24.0	3.01652	1.37986500396937
11	anger	N %	11.0	2.92232	3.07234252054536
12	consequence	N %	13.0	2.7172	2.02102534766134
13	observer	N %	9.0	2.57939	2.83440769591726
14	pain	N %	12.0	2.50461	1.85213597648825
15	doctor	N %	16.0	2.49418	1.4094525763272

**Table:** fear Verbs

#	Collocate	POS	*A*	TTEST	MI
1	express	V %	128.0	10.54204	3.87395526540072
2	allay	V %	61.0	7.78987	8.58183478778567
3	overcome	V %	49.0	6.65938	4.36112434268113
4	confirm	V %	45.0	5.78048	2.85415853515117
5	raise	V %	55.0	5.5502	1.99073126057856
6	live	V %	68.0	5.53562	1.60512345790394
7	tremble	V %	27.0	4.99968	4.72502063178775
8	grow	V %	42.0	4.42946	1.65963132681028
9	lose	V %	51.0	4.40847	1.38575080750967
10	dismiss	V %	25.0	4.38351	3.01976885081675
11	dispel	V %	16.0	3.91894	5.62486590864961
12	ease	V %	17.0	3.70883	3.31506846989762
13	paralyse	V %	14.0	3.66417	5.59364149991436
14	grip	V %	14.0	3.51272	4.03064939902881
15	conquer	V %	12.0	3.33719	4.77062459896117

**(4) ENGLISH ANGER**

**Table:** anger Adj

#	Collocate	POS	*A*	MI	TTEST
1	suppress	AJ %	12.0	4.85282571872024	3.43412
2	sudden	AJ %	16.0	7.98637993928282	3.3923
3	righteous	AJ %	10.0	7.82352946360078	3.12551
4	pent-up	AJ %	7.0	3.1310922345703	2.62304
5	frustrated	AJ %	7.0	2.5126359051346	2.53489
6	grow	AJ %	12.0	8.190738437789	2.37919
7	widespread	AJ %	8.0	7.48548170335014	2.0838
8	genuine	AJ %	8.0	5.90372875910487	2.07571
9	bitter	AJ %	7.0	3.68393979006403	2.06477
10	savage	AJ %	5.0	2.58947412473875	2.05493
11	simmer	AJ %	4.0	5.46874768759034	1.951
12	impotent	AJ %	4.0	3.22958936676036	1.92945
13	fierce	AJ %	5.0	4.12839894415525	1.78703
14	passionate	AJ %	4.0	2.04408675710745	1.70603
15	sullen	AJ %	3.0	3.04408675710745	1.64681

**Table:** anger Nouns

#	Collocate	POS	*A*	TTEST	MI
1	frustration	N %	28.0	5.27069	7.99038955253895
2	pain	N %	10.0	2.98635	4.16787102949495
3	fear	N %	10.0	2.9646	3.99974288348593
4	grief	N %	7.0	2.6095	6.18953034177411
5	sadness	N %	6.0	2.42786	6.8234922437942
6	guilt	N %	6.0	2.40267	5.70932122387489
7	love	N %	7.0	2.2727	2.82622261057866
8	environmentalist	N %	5.0	2.21612	6.8088568974258
9	resentment	N %	5.0	2.20327	6.09128690154808
10	despair	N %	5.0	2.19705	5.84056575715314
11	anxiety	N %	5.0	2.14034	4.5459158366822
12	desire	N %	5.0	2.06153	3.67931787923079
13	plan	N %	5.0	1.54821	1.70078704756518
14	decision	N %	5.0	1.4886	1.58087479439372
15	Mr	N %	5.0	0.64515	0.491107238190879

**Table:** anger Verbs

#	Collocate	POS	*A*	TTEST	MI
1	express	V %	66.0	7.74773	4.43221495690917
2	feel	V %	64.0	6.15543	2.11671363624743
3	vent	V %	25.0	4.99151	9.20196410557751
4	rise	V %	24.0	4.13709	2.68482441228638
5	provoke	V %	17.0	3.99844	5.04763040233376
6	control	V %	20.0	3.85721	2.8624896857616
7	direct	V %	17.0	3.79197	3.63825635473554
8	tremble	V %	15.0	3.78068	5.39088929738279
9	suppress	V %	13.0	3.50909	5.2241961291531
10	seethe	V %	12.0	3.45044	7.98672838735455
11	flush	V %	12.0	3.39574	5.66324146461063
12	boil	V %	12.0	3.37795	5.32952100216352
13	arouse	V %	12.0	3.36474	5.12364715326055
14	turn	V %	29.0	3.30523	1.37245215503444
15	explode	V %	11.0	3.19218	4.73615410250922

**(5) POLISH SMUTEK ‘sadness’**

**Table:** smutek ‘sadness’ Adjs

#	Collocate	POS	*A*	TTEST	MI	English equivalents
1	głęboki	adj	100.0	9.62014	4.718384525917518	deep
2	pełny	adj	74.0	7.08764	2.505712563981208	full
3	wielki	adj	140.0	6.70255	1.2057934153992946	great
4	przejmujący	adj	21.0	4.49927	5.781630909286284	piercing
5	bezbrzeżny	adj	20.0	4.46241	8.844940564569718	boundless
6	ogromny	adj	28.0	3.75628	1.7852289021035994	huge
7	jakiś	adj	68.0	3.75626	0.8770307726918485	some
8	nagły	adj	15.0	3.43592	3.1475205342043675	sudden
9	beznadziejny	adj	11.0	3.16989	4.498455365438376	hopeless
10	dziwny	adj	18.0	3.16796	1.9810526740358376	strange
11	nieokreślony	adj	11.0	3.1565	4.3724172957799885	indefinite
12	straszny	adj	14.0	3.08689	2.5146336578758195	terrible
13	twój	adj	28.0	3.07065	1.2525660262079732	your
14	szczery	adj	11.0	2.96564	3.2402250202761476	sincere
15	bezgraniczny	adj	9.0	2.95489	6.0552864308766505	total, boundless

**Table:** smutek ‘sadness’ Nouns

#	Collocate	POS	*A*	TTEST	MI	English equivalents
1	tropik	Noun	25.0	4.97285	7.524649762807013	tropic
2	emancypantka	Noun	10.0	3.14985	7.991593411377508	emancipation
3	rozstanie	Noun	9.0	2.66026	3.1424507042594403	parting, departure
4	trening	Noun	9.0	1.32917	0.8443968839372704	training
5	głowa	Noun	24.0	−3.91244	−0.846892874062491	head
6	życie	Noun	11.0	−28.51125	−3.262502915343643	life
7	pan	Noun	14.0	−64.37188	−4.186192169945479	lord, sir

**Table:** smutek ‘sadness’ Verbs

#	Collocate	POS	A	TTEST	MI	English equivalents
1	pogrążyć	Verb	104.0	10.15996	8.06501751021011	plunge
2	powiedzieć	Verb	104.0	6.82096	1.5944442024143786	say, perf.
3	ogarnąć	Verb	44.0	6.53829	6.126319255129692	overwhelm, perf.
4	czuć	Verb	59.0	6.49102	2.6902026984347276	feel
5	stwierdzić	Verb	49.0	6.1137	2.9814933148417393	state, perf.
6	ogarniać	Verb	36.0	5.93357	6.4970509794751035	overwhelm
7	stwierdzać	Verb	38.0	5.6447	3.568165293643454	state, perf.
8	mówić	Verb	117.0	5.60903	1.054556337395112	speak
9	patrzyć	Verb	44.0	5.47989	2.5238720909196486	look
10	napawać	Verb	30.0	5.44322	7.331627370267731	fill with
11	odczuwać	Verb	30.0	5.23914	4.5238736014930625	feel
12	pokiwać	Verb	26.0	5.05222	6.76757908543353	nod
13	topić	Verb	24.0	4.86758	7.285823680654605	sink
14	pomyśleć	Verb	31.0	4.86632	2.9887003218132326	think, perf.
15	poczuć	Verb	28.0	4.81192	3.4638219826326004	feel, perf.

**(6) POLISH RADOŚĆ ‘joy’**

**Table:** radość ‘joy’ Adjs

#	Collocate	POS	*A*	TTEST	MI	English equivalents
1	wielki	Adj	1211.0	29.06444	2.6012025050944603	great
2	ogromny	Adj	364.0	17.67869	3.768377577190495	huge
3	pełny	Adj	245.0	12.91525	2.5156060943006318	full
4	wieczny	Adj	136.0	11.19583	4.645093820893503	eternal
5	prawdziwy	Adj	158.0	9.9288	2.2508007593498993	true
6	mój	Adj	321.0	7.19571	0.7408796619335235	my
7	szczery	Adj	49.0	6.45318	3.678212202699862	sincere
8	nieopisany	Adj	32.0	5.60824	6.862552649224834	undescribable
9	spontaniczny	Adj	33.0	5.50789	4.601210707198511	spontaneous
10	jaki	Adj	263.0	5.44058	0.5896169130698841	what
11	wspólny	Adj	81.0	5.21569	1.2498953913064068	common
12	autentyczny	Adj	33.0	5.19278	3.380028405333701	authentic
13	niekłamany	Adj	26.0	5.05267	6.781632653841267	genuine, unfeigned
14	jakiż	Adj	28.0	4.9899	4.132966585424224	what
15	dziki	Adj	32.0	4.98768	3.079554440304421	wild

**Table:** radość ‘joy’ Nouns

#	Collocate	POS	*A*	TTEST	English equivalents	MI
1	życie	Noun	881.0	17.9874	life	1.3437726318852765
2	być	Noun	31.0	4.40434	be	2.258720377146277
3	granie	Noun	23.0	3.87483	playing	2.3805130933913694
4	istnieć	Noun	47.0	3.56654	exist	1.0595934265879448
5	macierzyństwo	Noun	16.0	3.33612	maternity	2.590995027829863
6	dawać	Noun	11.0	3.07789	give	3.796221775610776
7	obcowanie	Noun	12.0	2.8622	commune with	2.5248920077904597
8	zmartwychwstanie	Noun	12.0	2.61664	resurrection	2.031269818607729
9	przebywać	Noun	11.0	2.42724	be	1.898840577173269
10	odkrywać	Noun	9.0	2.39666	uncover	2.313924419910811
11	leń	Noun	7.0	2.30152	lazyboned	2.94220724727895
12	tworzyć	Noun	40.0	1.84586	create	0.497885623438381
13	niebo	Noun	44.0	1.62614	heaven	0.4057367698498833
14	sex	Noun	12.0	1.39106	sex	0.7407349083963752
15	kibic	Noun	30.0	1.1957	sports fan	0.3553210853880615
						0.45362758262754654

**Table:** radość ‘joy’ Verbs

#	Collocate	POS	*A*	TTEST	MI	English equivalents
1	sprawiać	Verb	504.0	22.08733	5.952141330543243	cause
2	sprawić	Verb	447.0	20.82657	6.06497899598923	cause, perf.
3	dawać	Verb	393.0	18.23574	3.641537326575362	give
4	przyjąć	Verb	203.0	12.05626	2.7007213612338243	receive
5	czerpać	Verb	149.0	11.99963	5.882389373724917	draw, take
6	przynosić	Verb	153.0	11.81848	4.4889955661110506	bring
7	wyrażać	Verb	134.0	10.97441	4.266579242474314	express
8	kryć	Verb	118.0	10.40984	4.583935563803693	hide
9	wyrazić	Verb	123.0	10.322	3.8510735420144098	express, perf.
10	przeżywać	Verb	113.0	10.188	4.5874882621293835	live through
11	zapanować	Verb	106.0	10.1409	6.056138762829899	control
12	ukrywać	Verb	111.0	9.9533	4.177236789131274	hide
13	dzielić	Verb	113.0	9.93135	3.927155415672382	share
14	odczuwać	Verb	105.0	9.82848	4.613937480496469	feel
15	witać	Verb	101.0	9.52409	4.256566312537501	greet

**(7) POLISH STRACH ‘fear’**

**Table:** strach ‘fear’ Adjs

#	Collocate	POS	A	TTEST	MI	English equivalents
1	blady	Adj	175.0	13.0097	5.916250334092935	pale
2	paniczny	Adj	156.0	12.46428	8.92411439987302	panic
3	ciągły	Adj	112.0	10.08541	4.410619730980793	continuous
4	paraliżujący	Adj	63.0	7.90518	7.9512505298105305	paralysing
5	wielki	Adj	289.0	6.72895	0.7269513218272461	great
6	zwierzęcy	Adj	38.0	5.8744	4.409768213512196	animal
7	zwykły	Adj	58.0	5.85945	2.116431208572637	usual
8	irracjonalny	Adj	29.0	5.27866	5.659998918648893	irrational
9	nagły	Adj	36.0	5.18837	2.8860701809104006	sudden
10	silny	Adj	58.0	4.9082	1.49198887479362	strong
11	oszalały	Adj	24.0	4.83715	6.308125003531237	mad
12	zabobonny	Adj	23.0	4.7656	7.309460214215058	superstitious
13	polny	Adj	25.0	4.68595	3.992842449031162	field
14	własny	Adj	124.0	4.61105	0.7712358127333814	own
15	ludzki	Adj	58.0	4.55647	1.315786141623697	human
16	wieczny	Adj	26.0	4.16641	2.4508769817106897	eternal

**Table:** strach ‘fear’ Nouns

#	Collocate	POS	*A*	TTEST	English equivalents	MI
1	gardło	Noun	7.0	−0.89811	throat	−0.42164320669473987
2	zwierzę	Noun	8.0	−11.04438	animal	−2.2941874842625167
3	serce	Noun	7.0	−17.56287	heart	−2.933221290580398
4	oko	Noun	25.0	−21.57289	eye	−2.4099552416192616
5	twarz	Noun	7.0	−22.2505	face	−3.234179425924676
6	śmierć	Noun	7.0	−27.53372	death	−3.5118181858857818
7	mieszkaniec	Noun	11.0	−30.41866	inhabitant	−3.3464705273192554
8	człowiek	Noun	17.0	−36.95889	human being	−3.316702864486338
9	kobieta	Noun	7.0	−51.02267	woman	−4.342324076374389
10	ludzie	Noun	22.0	−57.01557	people	−3.717622771413607
11	dziecko	Noun	18.0	−58.50026	child	−3.8864179165228476
12	nic	Noun	8.0	−66.75124	nothing	−4.620593769285852
13	co	Noun	9.0	−351.53801	what	−6.884833981019253

**Table:** strach ‘fear’ Verbs

#	Collocate	POS	A	TTEST	MI	English equivalents
1	pomyśleć	Verb	493.0	21.69759	5.455463088676508	think
2	czuć	Verb	258.0	14.42509	3.2943021453683787	feel
3	budzić	Verb	225.0	14.39148	4.623507549794386	wake
4	żyć	Verb	204.0	12.60056	3.0857761781248367	live
5	paść	Verb	166.0	12.37227	4.653797252825121	fall
6	trząść	Verb	136.0	11.55448	6.7624001168971555	shiver
7	odczuwać	Verb	135.0	11.29606	5.169313843807614	feel
8	drżeć	Verb	120.0	10.82621	6.4165104245294025	tremble
9	ogarniać	Verb	111.0	10.42682	6.597057085255134	overwhelm
10	umierać	Verb	111.0	10.23981	5.1542885757663095	die
11	poczuć	Verb	118.0	10.1907	4.0146253508090775	feel
12	sparaliżować	Verb	98.0	9.83392	7.238033012154485	paralyse, perf.
13	napędzić	Verb	94.0	9.68509	9.882968365928376	drive, increase
14	ogarnąć	Verb	96.0	9.61302	5.72736537808579	overwhelm
15	paraliżować	Verb	85.0	9.18782	8.183181598710803	paralyse
					5.4369804240091195	

**(8) POLISH GNIEW ‘anger 1’**

**Table:** gniew ‘anger 1’ Adjs

#	Collocate	POS	A	TTEST	English equivalents	MI
1	słuszny	Adj	59.0	7.46235	right, rational, justified	5.133641347994825
2	swój	Adj	186.0	6.22434	one’s	0.8793581420934464
3	boży	Adj	39.0	5.81522	godly	3.8610308496554664
4	bezsilny	Adj	32.0	5.60193	powerless	6.686423975874181
5	nagły	Adj	34.0	5.56835	sudden	4.4728081923281096
6	straszny	Adj	30.0	5.07263	terrible	3.7588847439086552
7	twój	Adj	37.0	4.3352	your	1.7993798822612401
8	srogi	Adj	18.0	4.18748	severe	6.2651274861236566
9	pełny	Adj	37.0	4.14513	full	1.650427976463129
10	klasowy	Adj	17.0	4.03112	class	5.486234655668294
11	niepohamowany	Adj	15.0	3.84909	irrepressible, uncontrollable	7.340772539466548
12	czerwony	Adj	19.0	3.58688	red	2.4972598404622723
13	straszliwy	Adj	14.0	3.5717	terrible	4.4604516642619325
14	pański	Adj	15.0	3.40364	lordly	3.0447439233724785
15	boski	Adj	15.0	3.39431	godly	3.0163455875091376

**Table:** gniew ‘anger 1’ Nouns

#	Collocate	POS	*A*	TTEST	MI	English equivalents
1	lud	Noun	47.0	6.30151	3.628971801934647	people
2	internauta	Noun	29.0	4.75976	3.106119253520865	Internaut
3	Bóg	Noun	47.0	2.84445	0.7732597322778273	God
4	niebiosy	Noun	7.0	2.53815	4.619951241351488	heavens
5	sprawiedliwy	Noun	8.0	2.44039	2.865724374349709	just, fair
6	ocean	Noun	8.0	2.16681	2.095924149866194	ocean
7	przeciwnik	Noun	7.0	0.12883	0.07201768826979889	opponent, enemy
8	Król	Noun	9.0	−0.3155	−0.1442629042229037	king
9	twarz	Noun	17.0	−0.90008	−0.28487133512225854	face
10	ojciec	Noun	20.0	−2.95814	−0.7324526211322907	father
11	rodzice	Noun	7.0	−5.01979	−1.5347104288155138	parents
12	głos	Noun	8.0	−12.78908	−2.4650919458017606	voice
13	ludzie	Noun	11.0	−24.12204	−3.048422599803925	people
14	Pan	Noun	23.0	−43.2737	−3.325269723464149	sir, lord

**Table:** gniew ‘anger 1’ Verbs

#	Collocate	POS	*A*	TTEST	MI	English equivalents	
1	wpadać	Verb	72.0	8.38549	6.409836889345482	fall into	
2	wywołać	Verb	48.0	6.65765	4.67849302335714	call forth	
3	wybuchać	Verb	41.0	6.35229	6.976716404244669	explode	
4	wyrażać	Verb	39.0	5.93832	4.347898726414907	express	
5	wpaść	Verb	39.0	5.88082	4.099966903839913	fall into, perf.	
6	wzbierać	Verb	34.0	5.81779	8.791443155281893	surge, rise	
7	wybuchnąć	Verb	35.0	5.76411	5.2827672339327645	explode, perf.	
8	czuć	Verb	48.0	5.73467	2.5372375622759638	feel	
9	wywoływać	Verb	35.0	5.69345	4.731923714020757	call forth	
10	bożyć	Verb	32.0	5.59483	6.511079824024953	God, verb	
11	budzić	Verb	35.0	5.43095	3.608209547131997	wake up	
12	ściągnąć	Verb	30.0	5.33645	5.282011667976065	pull down	
13	ogarnąć	Verb	29.0	5.27937	5.669584044101888	embrace, overwhelm	
14	unosić	Verb	27.0	5.06157	5.270891968813462	rise	
15	tłumić	Verb	26.0	5.06037		muffle, suppress	

**(9) POLISH ZŁOŚĆ ‘anger 2’**

**Table:** złość ‘anger 2’ Adjs

#	Collocate	POS	*A*	TTEST	MI	English equivalents
1	bezsilny	Adj	78.0	8.78705	7.625808557604507	powerless
2	sportowy	Adj	68.0	7.26377	3.069284862767255	sports
3	nagły	Adj	32.0	5.31281	4.039327713945847	sudden
4	czerwony	Adj	36.0	5.28712	3.0732396913290763	red
5	pełny	Adj	45.0	4.475	1.5868100700319316	full
6	swój	Adj	181.0	3.90101	0.49402758093669774	one’s own
7	wyraźny	Adj	15.0	3.09777	2.3207817702341016	clear
8	cały	Adj	87.0	3.00749	0.561573602491023	all
9	purpurowy	Adj	8.0	2.7568	5.303253340324792	purple
10	zapiekły	Adj	6.0	2.43906	7.876041127099847	(all) consuming
11	bezrozumny	Adj	5.0	2.21639	6.82834438506855	unreasonable, irrational, unjustified
12	blady	Adj	7.0	2.20803	2.5955766787959704	pale
13	niepohamowany	Adj	5.0	2.18347	5.40979240161347	uncontrollable
14	pomieszany	Adj	5.0	2.17521	5.19948545443459	mixed
15	agresywny	Adj	7.0	2.13047	2.36025611460128	aggressive

**Table:** złość ‘anger 2’ Nouns

#	Collocate	POS	*A*	TTEST	MI	English equivalents
1	piękność	Noun	10.0	2.87431	3.456980823403606	beauty
2	mama	Noun	13.0	−0.27559	−0.10626141709169179	mummy
3	twarz	Noun	11.0	−4.62061	−1.258920194867223	face
4	matka	Noun	14.0	−5.06752	−1.235330046011042	mother
5	ojciec	Noun	15.0	−7.03228	−1.493507757543057	father
6	nic	Noun	9.0	−23.21733	−3.1274862333657802	nothing
7	wszystko	Noun	15.0	−30.76678	−3.1609117650275143	all
8	pani	Noun	7.0	−32.4295	−3.728704112702629	lady
9	dziecko	Noun	7.0	−37.56426	−3.925805461429796	child
10	ludzie	Noun	7.0	−41.07343	−4.046516933515541	people
11	to	Noun	9.0	−113.83998	−5.283427668331679	this

**Table:** złość ‘anger 2’ Verbs

#	Collocate	POS	*A*	TTEST	MI	English equivalents
1	zrobić	Verb	154.0	10.93448	3.07248832910327	do, perf.
2	czuć	Verb	113.0	9.64141	3.4264363868380725	feel
3	robić	Verb	117.0	9.26836	2.8045002398402854	do, perf.
4	wpadać	Verb	56.0	7.33949	5.7012491728288515	fall
5	rzucić	Verb	55.0	6.96838	4.049697488096923	throw
6	poczuć	Verb	52.0	6.80649	4.1556045540660875	feel, perf.
7	wyładować	Verb	46.0	6.76473	8.590161504064355	vent, perf.
8	wyładowywać	Verb	43.0	6.5496	9.708592993764878	vent
9	ogarnąć	Verb	44.0	6.52408	5.925017030479689	overwhelm, perf.
10	pomyśleć	Verb	42.0	5.78788	3.225519209555116	think, perf.
11	wyrażać	Verb	38.0	5.76951	3.964406383864321	express
12	wpaść	Verb	38.0	5.69547	3.716474561289328	fall
13	trząść	Verb	33.0	5.65741	6.042513929483029	shiver
14	ogarniać	Verb	31.0	5.48546	6.080020063769664	overwhelm
					4.4600749005929945	

## Appendix 2

See Table 7.

**Table 7:** GRID features and their loadings on *valence*, *power*, *arousal* and *novelty* dimensions

Grid dimensions and features	Feature loadings
Valence dimension	
Frowned	0.962
Pressed lips together	0.921
Withdrew from people or things	0.882
Produced a short utterance	0.827
Felt cold	0.743
Produced speech disturbances	0.679
Had a trembling voice	0.643
Produced a long utterance	−0.667
Moved towards people or things	−0.837
Smiled	−0.967
Wanted to sing or dance	−0.975
Power dimension	
Had an assertive voice	0.908
Increased the volume of voice	0.799
Spoke faster	0.72
Spoke slower	−0.682
Closed his or her eyes	−0.684
Decreased the volume of voice	−0.732
Fell silent	−0.748
Arousal dimension	
Breathing getting faster	0.863
Heartbeat getting faster	0.832
Sweat	0.811
Perspired/moist hands	0.786
Felt hot	0.785
Felt shivers	0.701
Abrupt bodily movements	0.638
Heartbeat slowing down	−0.658
Breathing slowing down	−0.683
Novelty dimension	
Jaw dropped	0.82
Eyebrows went up	0.738
Opened his or her eyes widely	0.664

## Footnotes

1 Although we view the identification of the underlying reasons for cross-linguistic and cross-cultural differences in emotions as an important academic endeavour, it is beyond the scope and focus of the present paper.

2 An Application Programming Interface for the English version of the HASK dictionary of frequent word combinations was automatically generated from the British National Corpus [24].

3 The third *fear* scenario flight was also alluded to in [18].

4 However, recall that the present study only analyses GRID features pertaining to sensory cues. Using the full spectrum of power features Lewandowska-Tomaszczyk and Wilson [18] showed that *fear* and *strach* are characterised by *fight* and *fright*, respectively.
